# The synthesis of branched-chain fatty acids is limited by enzymatic decarboxylation of ethyl- and methylmalonyl-CoA

**DOI:** 10.1042/BCJ20190500

**Published:** 2019-08-30

**Authors:** Joseph P. Dewulf, Isabelle Gerin, Mark H. Rider, Maria Veiga-da-Cunha, Emile Van Schaftingen, Guido T. Bommer

**Affiliations:** 1 Laboratory of Physiological Chemistry, de Duve Institute, UCLouvain, Brussels B-1200, Belgium; 2 Walloon Excellence in Life Sciences and Biotechnology (WELBIO), UCLouvain, Brussels B-1200, Belgium; 3 Department of Laboratory Medicine, Cliniques Universitaires Saint-Luc, UCLouvain, Brussels B-1200, Belgium; 4 PHOS Laboratory, de Duve Institute, UCLouvain, Brussels B-1200, Belgium

**Keywords:** branched-chain fatty acids, ECHDC1, ethylmalonyl-CoA, fatty acid synthase, metabolite-repair, methylmalonyl-CoA

## Abstract

Most fatty acids (FAs) are straight chains and are synthesized by fatty acid synthase (FASN) using acetyl-CoA and malonyl-CoA units. Yet, FASN is known to be promiscuous as it may use methylmalonyl-CoA instead of malonyl-CoA and thereby introduce methyl-branches. We have recently found that the cytosolic enzyme ECHDC1 degrades ethylmalonyl-CoA and methylmalonyl-CoA, which presumably result from promiscuous reactions catalyzed by acetyl-CoA carboxylase on butyryl- and propionyl-CoA. Here, we tested the hypothesis that ECHDC1 is a metabolite repair enzyme that serves to prevent the formation of methyl- or ethyl-branched FAs by FASN. Using the purified enzyme, we found that FASN can incorporate not only methylmalonyl-CoA but also ethylmalonyl-CoA, producing methyl- or ethyl-branched FAs. Using a combination of gas-chromatography and liquid chromatography coupled to mass spectrometry, we observed that inactivation of ECHDC1 in adipocytes led to an increase in several methyl-branched FAs (present in different lipid classes), while its overexpression reduced them below wild-type levels. In contrast, the formation of ethyl-branched FAs was observed almost exclusively in ECHDC1 knockout cells, indicating that ECHDC1 and the low activity of FASN toward ethylmalonyl-CoA efficiently prevent their formation. We conclude that ECHDC1 performs a typical metabolite repair function by destroying methyl- and ethylmalonyl-CoA. This reduces the formation of methyl-branched FAs and prevents the formation of ethyl-branched FAs by FASN. The identification of ECHDC1 as a key modulator of the abundance of methyl-branched FAs opens the way to investigate their function.

## Introduction

Branched fatty acids (FA) are present in many organisms including mammals, usually as minor constituents and mostly in adipose tissues [1,2]. Most commonly, they carry methyl-branches close to the end of the carbon chain. This type of branched FAs is formed as a consequence of substrate promiscuity: FA synthase usually uses acetyl-CoA to start FA synthesis. Yet, instead of using its canonical substrate, it can use other CoA esters that carry methyl branches and are produced during the degradation of branched-chain amino acids [1,2]. Usage of these methyl-branched CoA esters leads to FAs with methyl groups on the penultimate and antepenultimate carbons, which are also called ‘iso' and ‘anteiso' monomethyl-branched FAs, respectively. Not surprisingly, they are observed in all life forms at low concentrations [3–5], but they can be the dominant FA type in certain bacteria [3]. Recently, this type of FAs has received renewed attention and it was found that their production in mammals is modulated by hypoxia and obesity [2]. Incorporation of these methyl-branched FAs in membrane lipids modifies membrane fluidity, lipid bilayer thickness and membrane-dependent functions such as oxidative phosphorylation in bacteria [6], but their function in mammals is largely unknown.

**Figure 1.**
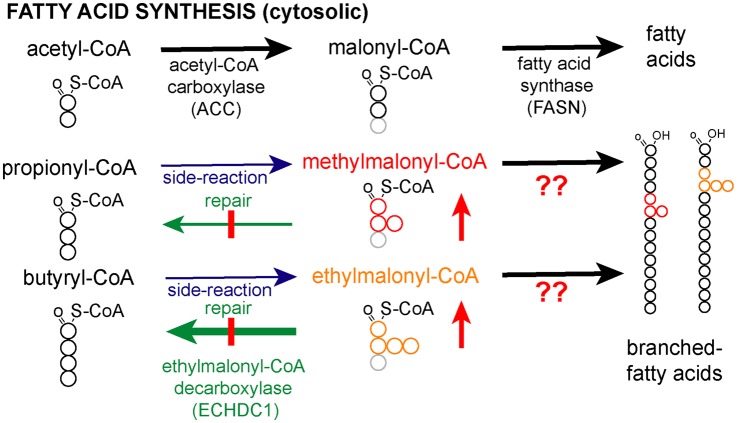
Does ECHDC1 prevent the synthesis of methyl- and ethyl-branched fatty acids? Acetyl-CoA carboxylase (ACC) catalyzes the synthesis of malonyl-CoA, which is used by fatty acid synthase for fatty acid (FA) extension of acetyl-CoA. Methylmalonyl-CoA and ethylmalonyl-CoA are also produced in the cytosol as side-products by ACC. Since ECHDC1 is known to be a (m)ethylmalonyl-CoA decarboxylase mainly localized in the cytosol, we hypothesized that the loss of this enzyme would lead to the formation of methyl- and ethyl-branched FAs by the incorporation of methylmalonyl-CoA and ethylmalonyl-CoA during FA synthesis.

Methyl branches may also be present at other places than the penultimate or antepenultimate carbons. This is the case for isoprenoid-derived lipids such as dolichol or farnesol but also diet-derived phytanic and pristanic acid. Besides, they can be synthesized due to dual substrate promiscuity of both acetyl-CoA carboxylase (ACC) and FA synthase (Figure 1). ACC carboxylates acetyl-CoA to synthesize malonyl-CoA, the major substrate used by FA synthase for chain elongation (Figure 1). In addition, it can use propionyl-CoA and even butyryl-CoA to synthesize methylmalonyl-CoA and ethylmalonyl-CoA, respectively [7] (Figure 1). FA synthase usually uses malonyl-CoA to extend FA chains. Yet, it can also use methylmalonyl-CoA, albeit at a much lower rate compared with malonyl-CoA [8–10]. This may lead to the formation of FAs with methyl branches on any of the even-numbered carbon atoms. In some birds, these FAs can accumulate to high levels [11–14], due to the action of a cytoplasmic enzyme that selectively decarboxylates malonyl-CoA, leaving methylmalonyl-CoA as the main substrate available for FA synthesis [9,15,16]. In contrast, little is known how the abundance of these methyl-branched FAs is regulated or limited in mammals. In addition, an in-depth analysis of the FA synthase reaction products when using methylmalonyl-CoA is lacking.

Mammals have specific pathways to degrade methyl-branched FAs using alpha-oxidation for 3-methyl-branched FAs and beta-oxidation for 2-methyl-branched FAs [17,18]. This might allow them to prevent the accumulation of methyl-branched FAs. To the best of our knowledge, ethyl-branched lipid species are quite rare in animals [19,20], and their mechanism of synthesis and degradation is unknown. Yet, their scarcity might indicate that cells possess mechanisms to limit their abundance.

We have recently discovered that the enzyme ECHDC1 catalyzes reactions that might serve to prevent the formation of branched FAs. This enzyme decarboxylates ethylmalonyl-CoA and, to a lesser extent, methylmalonyl-CoA [21]. We, therefore, hypothesized that it might serve as a metabolite repair enzyme [22–24] to remedy the two side activities of ACC (Figure 1). The reduction in cytosolic ethylmalonyl-CoA and methylmalonyl-CoA concentrations by ECHDC1 might be expected to prevent (or slow down) the formation of methyl- and ethyl-branched FAs by FA synthase (Figure 1). In the present study, we tested this hypothesis using a combination of *in vitro* approaches and adipocyte knockout (KO) models.

## Experimental

### Cell culture

3T3-L1 preadipocytes were a gift of O. A. MacDougald (University of Michigan) and L929 cells were a gift of T. Michiels (de Duve Institute, UCLouvain). 3T3-L1 and L929 cells were cultured at 37°C in a 10% CO_2_ atmosphere in DMEM containing 1 g l^−1^ of glucose (BioWhittaker, Lonza, Verviers, Belgium), 10% bovine calf serum (Hyclone SH30073.03, South Logan, Utah, U.S.A.), 2 mM l-glutamine and penicillin/streptomycin 100 U ml^−1^ (Life Technologies) and 1 mM pyruvate (Life Technologies).

Adipocyte differentiation was performed as described [25]*.* Briefly, cells that had been confluent in the presence of calf serum for 2 days (Day 0) were incubated in 10% fetal bovine serum (FCS, Biochrom S0115, Berlin, Germany), 1 µM dexamethasone (Sigma–Aldrich), 0.5 mM methylisobutylxanthine (Sigma–Aldrich), 1 µg ml^−1^ insulin (Sigma–Aldrich), and 5 µM troglitazone (Santa Cruz Biotechnology). On day 2, the cells were fed with 10% FCS medium containing 1 µg ml^−1^ insulin, and on day 4, the cells were re-fed with 10% FCS medium.

In the experiments where we used labeled substrates, methylmalonic acid (methyl-d3, Cambridge Isotope Laboratories), ethylmalonic acid (methyl-d3, Cambridge Isotope Laboratories) or propionic acid-3,3,3-d3 (Sigma–Aldrich) was added to a final concentration of 2 mM at days 2 and 4. Where indicated, vitamin B12 (Sigma–Aldrich) was added to the medium at day 0, 2 and 4 at a final concentration of 2.5 µM. HEK 293T cells (a gift from Eric Fearon, University of Michigan) used for transfections were cultured at 37°C in 10% CO_2_ in DMEM containing 1 g l^−1^ of glucose (BioWhittaker, Lonza, Verviers, Belgium), 10% heat-inactivated fetal bovine serum (F7524, Sigma–Aldrich), 2 mM l-glutamine and 100 U ml^−1^ penicillin/streptomycin (Life Technologies).

### Generation of CRISPR–Cas9 ECHDC1 knockout clones in 3T3-L1 and L929 cells

CRISPR/Cas9 plasmids driving the expression of single guide RNAs were generated to inactivate the gene coding for ECHDC1 [26]. Plasmids were prepared by ligating annealed pairs of primers (sequences below) into the vector pSpCas9(BB)-2A-Puro (PX459) (a gift from F. Zhang, Massachusetts Institute of Technology; Addgene's plasmid no. 48139) [27] digested by BbsI. *ECHDC1* was targeted in exon 5 before a highly conserved glutamate residue in the motif GGGA**E**FTT by one (in 3T3-L1) or two different (in L929) guide RNAs. The design of guide RNA was performed with the help of the Zhang lab website (http://crispr.mit.edu). Pairs of primers to generate the construct for guide RNA 1 (pJD1) were 5′-CAC CGG ACC AGA GCA ACA CTT ATT AA (s) and 5′-AAA CTT AAT AAG TGT TGC TCT GGT CC (as). Pairs of primers to generate the construct for guide RNA 2 (pJD2) were 5′-CAC CGG CAA GGC TGG GCA ATG GGT GG (s) and 5′-AAA CCC ACC CAT TGC CCA GCC TTG CC (as). Pairs of primers to generate the construct for guide RNA 3 (pJD16) were 5′-CAC CGG TTA CTA CAG CAT GTG ATT TC (s) and 5′-AAA CGA AAT CAC ATG CTG TAG TAA CC (as). All constructs were validated by sequencing.

3T3-L1 and L929 cells were transfected with CRISPR/Cas9 constructs using FuGene HD transfection reagent (Promega) according to the manufacturer's protocol. Following a transient selection with puromycin (ThermoFisher Scientific, 3 µg ml^−1^) for 48 h, cells were microdiluted in 96-well plates in order to isolate clonal populations. Genomic DNA was extracted and used to PCR-amplify the regions surrounding the targeted sites. PCR products were analyzed by Sanger sequencing (Genewiz). The clones used in this study had the following mutations:

3T3-L1 clone 1/JD50D (Target 1): change of reading frame caused by a deletion of 28 nt on allele 1 and a deletion of 4 nt on allele 2.

3T3-L1 clone 2/JD175-5 (Target 2): change of reading frame caused by a deletion of 1 nt on allele 1 and a large deletion on allele 2.

L929 clone 1/JD51 (Targets 1 and 3): change of reading frame caused by a deletion of 11 nt on allele 1 and of 1 nt on allele 2 (leading to a premature stop codon after 166 amino acids).

L929 clone 2/JD52 (Targets 1 and 3): change of reading frame caused by a deletion of 11 nt on allele 1 and of 1 nt on allele 2 (different from clone 1, this one led to a premature stop codon after 140 amino acids)

### Cloning of ECHDC1 and lentiviral transduction on cells

The mouse ECHDC1 open reading frame was obtained as a geneblock from Integrated DNA Technologies. Some preliminary experiments indicated the possibility of cryptic splice sites in the cDNA. Thus, the geneblock was ordered in a way that changed the nucleotide sequence, while maintaining the coded amino acid sequence (5′-A TAC ATG CTA GCC ACC ATG CGG AGA TGC GAA GTA AAC TCC AAG CCT ATA AGC GAA TAC TTC GGC ATT CCT TGT GAG AAT AGG GAA ATG GCA AAA TGT CTT CTT ACC TCC TCA CTC TCA GTA CGG ACT AAG CTG CTG CAA ACC GGC GTG TCA CTC TAC AAT ACA TCA CAT GGA TTC CAC GAA GAA GAA GTT AAA AAA ATC CTG GAG CAA TTC CCT GGT GGT TCC ATT GAT CTC CTG AAA AAA CAA AAC GGG ATA GGA ATA CTT ACA CTG AAC AAT CCC AAT AAG ATG AAT GCC TTT TCC GGC GTC ATG ATG CTG CAA TTG CTG GAA CGA GTG ATT GAG TTG GAA AAT TGG ACC GAA GGC AAA GGC CTT ATT ATT CAC GGA GCA AAG AAC ACT TTT TGT TCA GGG TCC GAC CTT AAT GCC GTA AAA GCC CTC AGT ACT CCA GAA AGC GGT GTC GCC CTC AGT ATG TTT ATG CAG AAT ACT CTC ACT CGC TTC ATG CGC CTT CCC CTT ATT TCC GTT GCA CTT GTG CAG GGC TGG GCT ATG GGA GGA GGG GCT GAA TTG ACC ACT GCC TGT GAT TTT AGA TTG ATG ACC GAG GAA TCT GTG ATA CGG TTC GTA CAC AAA GAA ATG GGG ATT GTA CCT AGT TGG GGG GGT ACT TCC CGC CTC GTC GAG ATC ATC GGG TCT CGG CAG GCC CTG AAG GTC CTT TCT GGG ACT CTC AAA CTG GAT AGC AAA GAG GCA CTT AAT ATC GGG CTG ACT GAC GAA GTG CTG CAA CCA TCC GAT GAG ACC ACC GCT TTG GAG CAG GCT CAG GAG TGG TTG GAG AAG TTC GTT TCC GGG CCA CCT CAG GTA ATC CGA GGC CTG AAA AAG TCC GTT TGT TCT GCT CGC GAA CTG TAT ATA GAG GAA GCT CTC CAG AAT GAA AGG GAT GTG TTG GAG ACT CTG TGG GGC GGG CCC GCA AAC CTG GAG GCT ATC GCC AAA AAA GGA AAG CAT ACA AAG TAG TGT ACA TTA TTT). The geneblock was amplified by PCR and inserted between XbaI and BamHI restriction sites of pMB1, a plasmid derived from lentiCRISPR V2 (a gift from Feng Zhang, Addgene plasmid # 52961) [28], in which we have introduced a guide RNA targeting lacZ [29]. The primers used to insert lacZ guide RNAs after BsmBI digestion were the following: 5′-CAC CGG CCC GAA TCT CTA TCG TGC GG (s) and reverse 5′-AAA CCC GCA CGA TAG AGA TTC GGG CC (as). The primers used to insert ECHDC1 geneblock were the following: sense 5′-ATA CAT **GCT AGC** CAC CAT GCG GAG AT (containing a NheI restriction site in bold) and reverse 5′-TTA TAT **GGA TCC** CTT TGT ATG CTT TCC TTT TTT GGC (containing a BamHI restriction site in bold). The resulting plasmid containing the ECHDC1 geneblock was named pJD219. It expresses ECHDC1 protein in frame with a puromycin resistance cassette separated by a self-cleaving P2A sequence [26].

Recombinant lentiviruses were produced by the transient transfection of the lentiviral vector and the packaging plasmids pMD2 and psPAX2 (both gifts from Didier Trono, Addgene, number 12259 and 12260, respectively) in HEK 293 T using polyethylenimine (jetPEI, Polyplus transfection). After incubation for 48 h at 37°C, the virus-containing medium of the cells was harvested, filtered (0.45 µm) and used to infect target cells in the presence of 8 µg ml^−1^ polybrene (Sigma–Aldrich). Selection of infected cells was performed with 3 µg ml^−1^ of puromycin for three days. L929 ECHDC1 KO clone 1 (JD51) and 3T3-L1 ECHDC1 KO clone 1 (JD50D) were infected with pMB1 as control or with pJD219 for the rescue, and renamed JD361 (L929 control), JD362 (L929 rescued), JD363 (3T3-L1 control) and JD364 (3T3-L1 rescued).

### Sample preparation and extraction of metabolites from adherent cells

Seven days after the induction of adipogenesis, the medium was removed, plates were rapidly washed with ice-cold water and plunged in liquid nitrogen to quench metabolic activity [30]. Metabolites were extracted using a method adapted from [31]*.* The frozen dishes were placed on crushed ice, and 300 µl cold methanol (MS-grade, Biosolve) was immediately added, followed by 250 µl of cold water. The cells were scraped and collected in 2 ml tubes containing 1 ml of tert-butyl methyl ether (Sigma–Aldrich). Samples were mixed and kept at least 4 h at −20°C before centrifugation at 16 000×***g*** for 10 min at 4°C. The upper layer contains the organic phase, while the lower layer contains aqueous metabolites and the pellet is composed of proteins and insoluble matrix. The lower aqueous layer was dried out and resuspended in methanol : water (1 : 1 v : v) before LC–MS analysis. The upper organic layer was dried down under a gentle stream of nitrogen in glass tubes at 40°C, and used for transesterification prior to GC–MS analysis or for LC–MS lipidomic analysis.

### Fatty acid synthase assays

Rat liver fFA synthase was purified from rat livers, as described in [32,33]. Assays were performed at 30°C by adding purified FA synthase to 1 ml of 50 mM phosphate buffer pH 7.2, 0.5 mg ml^−1^ bovine serum albumin (FA free, Sigma–Aldrich), 1 mM DTT (Sigma–Aldrich), 200 µM NADPH (Sigma–Aldrich) and 50 µM d3-acetyl-CoA. Malonyl-CoA (CoALA Biosciences) with or without the indicated concentration of methylmalonyl-CoA (CoALA Biosciences) or ethylmalonyl-CoA (CoALA Biosciences) was added and NADPH utilization was followed at 340 nm over 40 min. Assays used for GC–MS and LC–MS analysis were performed in 200 µl and incubation was for 6.5 h. Experiments were performed five times on separate days over a time period of 1 year for NADPH consumption assays and four times on separate days over a time frame of 1 month for GC and LC assays.

### Sample preparation and extraction of metabolites from fatty acid synthase assays

After 6.5 h incubation, samples were divided in two 100 µl aliquots. One was directly dried down and FA methyl esters were generated using a method described below. The second aliquot was heated for 30 min at 80°C after the addition of 1.5 µl of K_2_CO_3_ (Merck), dried down under vacuum and resuspended in methanol prior to LC/MS analysis.

### Release of fatty acids from the organic layer

To release FAs from the organic layer, we adapted a method described by [34]. Briefly, 200 µl of organic fraction (obtained during tert-butyl methyl ether extraction) was evaporated under nitrogen and the dry residue was resuspended in 500 µl of acetonitrile/water 9 : 1 (v : v) containing 0.5 M HCl. Hydrolysis was performed at 100°C in stoppered glass tubes for 2 h. One milliter of water and 1 ml of chloroform (spectro grade, ACROS Organics) were added before mixing and centrifugation at 930×***g*** for 2 min. The organic (lower) layer was dried down under nitrogen. Released FAs were resuspended in 50 µl of methanol before LC–MS analysis. Experiments were performed three times on separate days over a time frame of 2 months. In each experiment, three wells of a six-well plates were quenched and extracted separately for each condition. Each sample was analyzed separately (i.e. the samples were not pooled) by LC/MS.

### GC–MS analysis of fatty acids

GC–MS analysis was performed using an Agilent 7890A apparatus equipped with a 30 m DB-5 ultra-inert capillary column connected to an Agilent 5977 mass detector acquired in combined Selected-Ion Monitoring (SIM) and scanning mode. The method was adapted from [35], with a helium flow rate of 1.5 ml min^−1^.

For the analysis of fatty acid methyl esters (FAMEs), the set-up was as follows: After 2.5 min at 100°C, the oven temperature was ramped from 100°C to 175°C at a rate of 50°C min^−1^, held at 175°C for 20 min, ramped from 175°C to 225°C at a rate of 5°C min^−1^, held at 225°C for 3 min, ramped from 225°C to 310°C at a rate of 30°C min^−1^ and held at 310°C for 6 min. The following SIM chromatograms were recorded: m/z 88, 102, 186, 200, 214, 228, 242, 256, 270, 284, 298, 312, 326, 340, 354, 368, 382 and 396. When using deuterated substrates, we added m/z 245, 248, 251, 254, 259, 262, 265, 268, 273, 276, 279, 282, 285, 286, 287, 290, 293, 296, 299, 300, 301, 304, 307, 310, 313, 315, 316, 318, 321, 324, 329, 332, 335 and 338.

For the analysis of picolinyl-derivatives, the oven temperature was held 0.5 min at 100°C, ramped from 100°C to 225°C at a rate of 50°C min^−1^, held at 225°C for 25 min, ramped from 225°C to 300°C at a rate of 5°C min^−1^ and held at 300°C for 3 min. The following SIM chromatograms were recorded: 92, 108, 151, 164, 178, 192, 206, 220, 234, 235, 238, 248, 249, 262, 263, 266, 276, 277, 290, 291, 294, 304, 305, 318, 319, 332, 333, 336, 346, 347, 349, 350, 360, 361, 363, 364, 374, 375, 378, 388, 389, 392, 403 and 407.

Experiments were performed three times on separate days over a time frame of several weeks. In each experiment, three wells of a six-well plate were quenched, extracted and transesterified separately for each condition. Each sample was analyzed separately (i.e. the samples were not pooled) by GC/MS. In each experiment, the mean values for the normalized metabolite concentrations were calculated. The resulting three mean values (obtained in independent experiments) were then used to calculate means and SEM values.

### Fatty acid methyl ester transesterification

To prepare methyl esters from the dry residue after tert-butyl methyl ether extraction (from cells or FA synthase assays), we used an acid-catalyzed transesterification process [36] as follows: 100 µl of toluene (Sigma–Aldrich) was added to dissolve non-polar lipids prior to the addition of 500 µl of methanol and 150 µl of ‘methylester reagent' (8% HCl (Merck) in methanol). The reaction mixture was stoppered and kept at 50°C overnight. Finally, 1 ml of water was added and two n-hexane (VWR chemicals) extractions were performed by the addition of 2 ml and 1 ml of n-hexane, followed by centrifugation at 930×***g*** for 2 min. Pooled hexane fractions were dried down under nitrogen and reconstituted in 100 µl of n-hexane before the GC/MS analysis.

### 3-Pyridylcarbinol (‘picolinyl') ester transesterification of fatty acids

We adapted a method described by [37]. After tert-butyl methyl ether extraction, the organic layer was collected and passed through sodium sulfate drying cartridges (Agilent Technologies, Anhydrous Sodium sulf LRC 1 gm, 12131033) before being evaporated to dryness under nitrogen. The residue was dissolved in 1 ml dichloromethane (Sigma–Aldrich) and 300 µl of freshly prepared ‘picolinyl reagent' [2 : 1 v : v 3-pyridinemethanol (Sigma–Aldrich)/potassium tert-butoxide (Sigma–Aldrich)] was added for reaction in a stoppered glass tube for 30 min at 37°C. After cooling to room temperature, two steps of n-hexane extraction were carried out (as described above). Pooled organic fractions were dried through sodium sulfate drying cartridges and evaporated to dryness under nitrogen. The residue was reconstituted in 100 µl of hexane, and analyzed by GC–MS.

### Chemical synthesis of d3-acetyl-CoA

The method was adapted from [38]. Briefly, acetic anhydride-d6 (Sigma–Aldrich) was added to a cold solution of 0.5 M NaHCO_3_ (Merck)/Coenzyme A sodium salt hydrate (Sigma–Aldrich) and was kept on ice for 20 min. Formic acid (MS-grade, Biosolve) was then added until pH 3 was reached. The absence of residual free coenzyme A was verified by LC–MS-qTOF.

### LC–MS analysis

LC–MS analysis of aqueous samples extracted from cells, of free FAs from the FA synthase assays or of organic fractions obtained from cells was performed using a LC–MS qTOF scanning m/z between 69 and 1700, as described in [39]*.* Briefly, 5 µl of sample was injected and subjected to ion pairing chromatography with an Inertsil 3 µm particle ODS-4 column (150 × 2.1 mm, GL Biosciences) on an Agilent 1290 HPLC system using hexylamine (Sigma–Aldrich) as the pairing-agent. The flow rate was constant at 0.2 ml min^−1^ using mobile phase A (5 mM hexylamine adjusted to pH 6.3) and B (90% methanol/10% 10 mM ammonium acetate (Biosolve) adjusted to pH 8.5). For the detection of CoA esters, an Agilent 6550 ion funnel mass spectrometer used in the negative mode with an electrospray ionization (ESI) (voltage 3500 V, sheath gas 350°C at 11 L min^−1^, nebulizer pressure 35 psig and drying gas 200°C at 14 L min^−1^). The solvent gradient was: 0–2 min at 0% B; 2–6 min from 0 to 20% B; 6–17 min from 20 to 31% B; 17–36 min from 31 to 60% B; 36–41 min from 60 to 100% B; 41–51 min at 100% B; 51–53 min from 100 to 0% B; 53–60 min at 0% B. Experiments were performed three times on separate days over a time frame of 2 months. In each experiment, three wells of a six-well plate were quenched and extracted separately for each condition. Each sample was analyzed separately (i.e. the samples were not pooled) by LC–MS.

For the analysis of released FAs, the following solvent gradient was used : 0–2 min at 0% B; 2–5 min from 0 to 80% B; 5–8 min 80% B; 8–18 min from 80 to 90% B; 18–27 min 90% B; 27–35 min from 90% to 100% B; 35–44 min 100% B; 44–45 min from 100% B to 0% B; 45–50 min 0% B.

### Note about the analysis of fatty acid profiles by GC–MS or LC–MS

GC–MS analysis (of FA methyl esters) allowed a much better chromatographic resolution of the diverse stereoisomers carrying methyl-groups at different positions than the LC–MS analysis of FAs. Unfortunately, when we analyzed purified methyl-branched FA standards (as methyl esters) by GC–MS, we realized that signals were much lower for branched FAs than for their isomeric straight chain counterparts. Likewise, signal intensities for shorter chain FAs were much lower than those for longer chain FAs. We believe that this reduction in signal intensity is most likely a consequence of the higher volatility of branched and short FAs, which makes that they are lost during the preparation of FA methyl esters prior to GC–MS analysis. Given that we do not have standards for all possible stereoisomers, the quantitative comparison of branched and straight chain FAs by GC–MS is difficult, especially for those with the shortest straight chains or the polymethylated branched FAs. Yet, it leaves qualitative interpretations unaffected.

### LC–MS lipidomic analysis

Prior to the lipidomic analysis by LC–MS, 200 µl organic layers (tert-butyl methyl ether extraction) were resuspended in 100 µl methanol : isopropanol (1 : 1 v : v). Lipidomic profiling was performed using the same MS qTOF (Agilent 6550 ion funnel) settings as described above and scanning m/z between 69 and 1700, but the ESI source was used in both negative and positive modes.

Preliminary experiments had revealed that fragmentation techniques on our mass-spectrometer (Agilent 6550 qTOF and Lumos Fusion Tribrid) did not allow us to distinguish between straight chain FAs with an odd carbon number or the isomeric methyl-branched counterpart. To maximize the temporal resolution, we, therefore, only used MS1-acquisition.

The chromatography method was adapted from Thermo Fischer Scientific^TM^ ‘*Application note 648: Increased throughput and confidence for lipidomics profiling using comprehensive HCD MS^2^ and CID MS^2^/MS^3^ on a Tribrid Orbitrap mass spectrometer*'. The LC separation used two mobile phases at a flow rate of 0.260 ml min^−1^. Buffer A was composed of 10 mM NH_4_ formate, 0.1% formic acid in 60 : 40 (v : v) acetonitrile(MS-grade, Biosolve):water, while buffer B was composed of 10 mM ammonium formate (MS-grade, Biosolve), 0.1% formic acid in 90 : 10 (v : v) isopropanol (MS-grade, Biosolve) : acetonitrile. A total of 5 µl samples were analyzed on a Thermo Scientific^TM^ Accucore^TM^ C18 column (150 mm × 2.1 mm, particle size 2.6 µm) operated at 45°C using the following gradients: 0–2 min 30% B; 2–2.1 min 55% B; 2.1–12 min 65% B; 12–18 min 85% B; 18–20 min 100% B; 20–30 min 100% B; 30–30.1 min 30% B; 30.1–35 min 30% B.

Experiments were performed three times on separate days over a time frame of 2 months. In each experiment, three six-well plates were quenched and extracted separately for each condition. Each sample was analyzed separately (i.e. the samples were not pooled) by LC–MS and injected twice: once in ESI negative mode and once in ESI positive mode. In each experiment, the mean values for the normalized metabolite concentrations were calculated. The resulting three mean values (obtained in independent experiments) were then used to calculate the means and SEM values.

### Quantification and statistical analysis

GraphPad Prism (version 8.0.2) and Microsoft Excel were used to perform statistical analyses. All data (normalized metabolites measured by GC–MS or LC–MS) were log-transformed before statistical analysis and the presence of a normal distribution was assessed for straight chain FA abundances. One-way ANOVA was performed for multiple group comparisons. Post hoc testing was performed using Dunnett's test when compared to a single control group, while Tukey's test was used when comparing several pairwise groups. When two groups were compared, we performed multiple paired *t*-tests and corrected for multiple testing using the Benjamini Hochberg approach [40]. A corrected *P* value <0.05 was considered significant and indicated by an asterisk in the figures.

## Results

### Fatty acid synthase can use methylmalonyl-CoA to make several methyl-branched fatty acids

We first wanted to confirm that FA synthase can use substrates other than malonyl-CoA during FA chain extension, and characterize the resulting products. Therefore, we set up an *in vitro* reaction with purified rat FA synthase, where we followed the consumption of NADPH in a spectrophotometric assay. To characterize the products, we used deuterated acetyl-CoA (d3-acetyl-CoA) as a starting unit to facilitate the interpretation of mass-spectrometrical analyses (which otherwise is confounded by FAs contaminating albumin).

**Figure 2.**
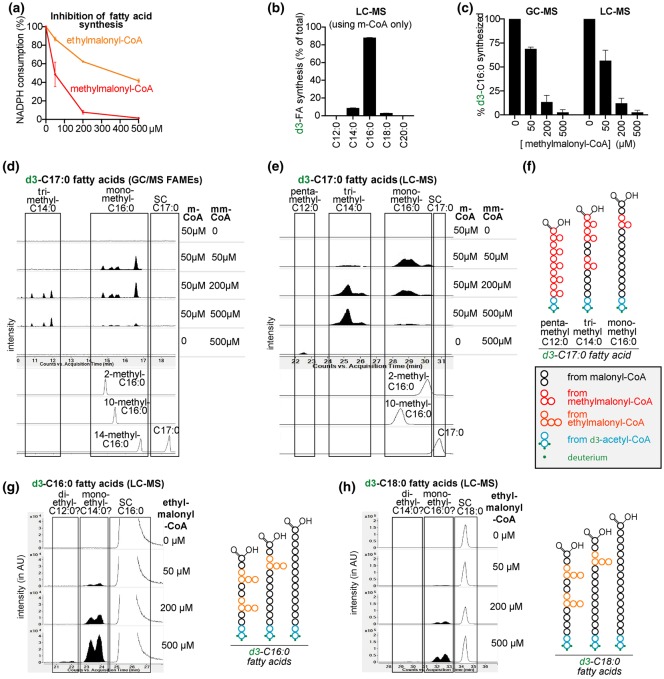
Fatty acid synthase can synthesize fatty acids with methyl- or ethyl-branches on even-numbered carbon atoms. (**a**) The activity of purified rat liver fatty acid synthase was determined in the presence of 50 µM malonyl-CoA (m-CoA) and increasing concentrations of (m)ethylmalonyl-CoA by monitoring NADPH consumption at 340 nm. Data shown are means ± SD of two experiments and are normalized to the activity when only malonyl-CoA was present. Comparable results were obtained in three additional experiments. (**b** and **c**) Quantification of the indicated fatty acids (FAs) produced in 6.5 h by FA synthase using malonyl-CoA and d3-acetyl-CoA ± methylmalonyl-CoA (mm-CoA) as indicated. (**d** and **e**) Similar reactions were analyzed by GC–MS (as fatty-acid methyl esters, m/z 287) (**d**) and LC–MS, m/z 272.26745 (**e**). Representative extracted ion chromatograms (EIC) of m/z corresponding to FAs with 17 carbons are presented. The tentative assignment as mono-, tri- and penta-methyl FAs is indicated. The elution profiles of three monomethyl-C16 : 0 FA standards are also shown. (**f**) Schematic representation of monomethyl-C16 : 0, trimethyl-C14 : 0 and penta-methyl-C12 : 0 branched FAs with 17 total carbons, which can be formed in the reactions analyzed in (**d** and **e**). (**g** and **h**) Representative EICs corresponding to deuterated FAs with 16 (**g**) and 18 (**h**) total carbons (the left part of the panels), m/z 258.2518 and 286.2831, respectively. On the right, schematic representation of monoethyl- and diethyl-FAs with 16 and 18 carbons. SC, straight chain. AU, arbitrary units.

The consumption of NADPH was easily detectable in the presence of 50 µM malonyl-CoA, but remained below the detection limit in the presence of 500 µM methylmalonyl-CoA or ethylmalonyl-CoA alone (data not shown). Yet, the activity in the presence of 50 µM malonyl-CoA was inhibited by 50% when methylmalonyl-CoA and ethylmalonyl-CoA were present at 50 and 500 µM, respectively (Figure 2a). This indicated that methyl- and ethylmalonyl-CoA can bind to FA synthase, even though their usage in FA elongation was below the detection limit of the spectrophotometric assay. These results also show that the affinity of FA synthase is ∼10-fold higher for methylmalonyl-CoA than for ethylmalonyl-CoA.

In a second step, we wanted to determine with a more sensitive technique whether FA synthase can use ethyl- or methylmalonyl-CoA. To this end, we incubated *in vitro* reactions identical with the ones described above for a longer time (6.5 h) and analyzed the produced FAs by GC–MS and LC–MS. When we analyzed reactions containing only malonyl-CoA, we observed that mainly straight chain C16 : 0 was produced (Figure 2b) while C14 : 0 and C18 : 0 were much lower and branched FAs were absent.

The addition of methylmalonyl-CoA to malonyl-CoA-containing reactions provoked a dose-dependent decrease in the formation of straight chain FAs (Figure 2c. showing results for C16 : 0), consistent with the observed overall inhibition of FA synthase (Figure 2a). This was accompanied by the appearance of putative methyl-branched FAs, which in GC–MS and LC–MS eluted slightly before their isomeric straight chain counterparts (e.g. monomethyl-C16 : 0 species before straight chain C17 : 0) (Figure 2d,e), as previously described [41]. Methylmalonyl-CoA incorporation during FA elongation leads to methyl branches on any even-numbered carbon atom except the last one, explaining the presence of multiple peaks. Methyl-branched FAs with a total number of carbons ranging from 15 to 18, and from 8 to 21 were observed in GC–MS (Supplementary Fig. S1b–d) and LC–MS analysis (Supplementary Fig. S1e–r), respectively. Using synthetic monomethyl-branched C16 : 0 FAs as standards, we were able to delineate the elution times of several 17-carbon branched FAs (Figure 2d,e, lower part). In addition, we observed one (GC–MS) or two (LC–MS) additional groups of 17 carbon FAs, which eluted earlier and were more efficiently produced with increasing methylmalonyl-CoA concentrations (Figure 2d,e). Utilization of three methylmalonyl-CoA and five methylmalonyl-CoA molecules during FA elongation can also lead to the synthesis of FAs containing 17 carbons (Figure 2f). This suggests that the earlier eluting 17-carbon FAs correspond to tri- and penta-methyl FAs (Figure 2d,e). Of note, 17 carbon FAs with two or four methyl branches cannot be synthesized under these conditions since the synthesis starts with acetyl-CoA and an incorporation of an even number of methylmalonyl-CoA molecules would inevitably lead to FAs with an even number of carbons (Supplementary Fig. S1a). Following a similar line of reasoning, two groups of FAs with 18 carbons were deemed to be tetramethyl-branched C14 : 0 (eluting first) and dimethyl-branched C16 : 0 (Supplementary Fig. S1d,j). Comparable observations were made for other methyl-branched FAs (Supplementary Fig. S1b–r).

With increasing concentrations of methylmalonyl-CoA, overall FA production was reduced, indicating that the incorporation of methylmalonyl-CoA is much less efficient than that of malonyl-CoA (Figure 2c and Supplementary Fig. S1). In parallel, we noted that concentrations of methylmalonyl-CoA beyond 200 µM decreased the formation of mono- and di-methyl branched FAs, while the formation of polymethylated-branched FAs continued to increase (Supplementary Fig. S2). This was expected, since the formation of di- and trimethyl-branched FAs is more likely to occur with higher concentrations of methylmalonyl-CoA (Figure 2d,e and Supplementary Fig. S2). When only methylmalonyl-CoA was present in the incubation mixture, we expected that each elongation reaction would introduce a methyl branch. Consistent with this, we observed the production of FAs that were deemed to contain methyl groups on all even-numbered carbons (i.e. dimethyl-branched C6 : 0, trimethyl-branched C8 : 0, tetramethyl-branched C10 : 0, pentamethyl-branched C12 : 0 and hexamethyl-branched C14 : 0 in Figure 2e and Supplementary Fig. S1) on the basis of their total number of carbons and their relative elution time.

Altogether, we conclude that FA synthase can use methylmalonyl-CoA during FA elongation and this leads to the incorporation of methyl-branches at different positions. Up to six methyl branches can be incorporated leading to methyl-branches on each even-numbered carbon (Supplementary Fig. S1k).

### Evidence for a weak incorporation of ethylmalonyl-CoA by fatty acid synthase

When we analyzed FA synthase reactions containing ethylmalonyl-CoA by LC–MS, we detected putative monoethyl- and diethyl-branched FAs in reaction mixtures containing ethylmalonyl-CoA together with malonyl-CoA. As for methyl-branched FAs, the peaks preceded their isomeric straight chain counterparts (Figure 2g,h and Supplementary Fig. S3). For the putative monoethyl-branched FAs, the signal was proportional to the concentration of ethylmalonyl-CoA used in the reactions while in the case of the putative diethyl-branched FAs, a sigmoidal dependency was observed, in agreement with the incorporation of two molecules of ethylmalonyl-CoA (Supplementary Fig. S3h).

The relative signal intensity of the putative monoethyl-branched FA in comparison with its straight chain counterpart was 20 times less than what we observed for methyl-branched FAs in incubations with methylmalonyl-CoA. This might explain why we were not able to detect ethyl-branched FAs using GC–MS, since this approach has lower sensitivity and suffers from the loss of more volatile FAs during the derivatization process (see Methods). Furthermore, ethyl-branched FAs partially coelute with (more abundant) methyl-branched FAs (see also later in Figure 7b).

We conclude that FA synthase can incorporate ethylmalonyl-CoA to make ethyl-branched FAs, albeit with an efficiency that is much lower than the formation of methyl-branched FAs using methylmalonyl-CoA.

### ECHDC1 knockout cells accumulate fatty acids with methyl-branches on all even-numbered carbons

To investigate the role of ECHDC1 in preventing branched FA synthesis, we used both the mouse preadipocyte cell line 3T3-L1, which has been extensively used for FA metabolism [2], and mouse L929 cells, which are also able to differentiate into adipocytes [42] using a protocol commonly used to differentiate mouse embryonic fibroblasts into adipocytes [43]. The second model shows less clonal variability in their potential to differentiate into adipocytes.

**Figure 3.**
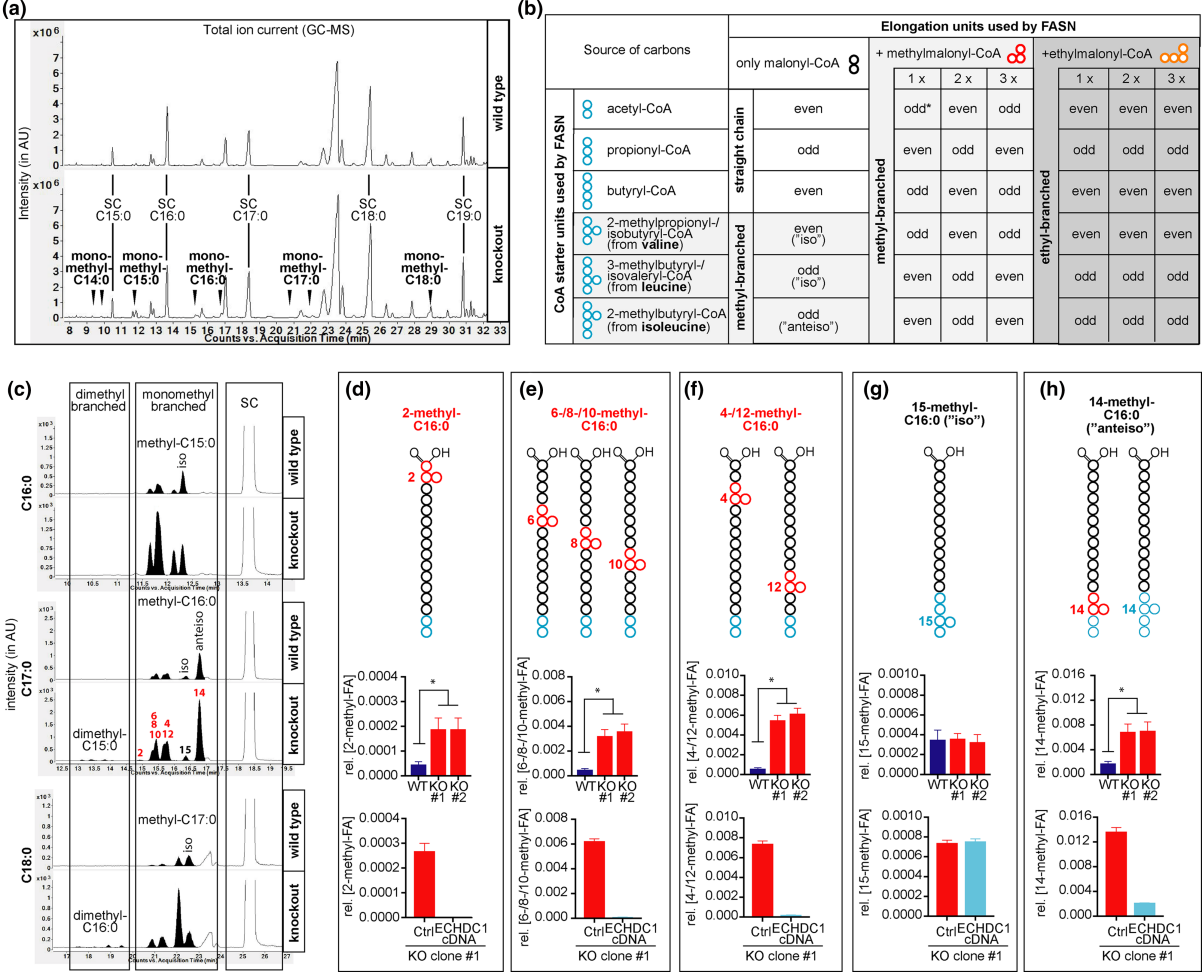
ECHDC1 limits but does not completely prevent the formation of methyl-branched fatty acids on even carbons in adipocytes. (**a**) GC–MS profiles of fatty acid methyl esters (FAMEs) derived from wild-type (WT) or ECHDC1 knockout (KO) L929 cells. Straight chain (SC) fatty acids (FAs) and putative branched Fas (arrowheads) are indicated in the total ion chromatograms (TICs). (**b**) Table representing the possibilities to form straight or branched FAs by FA synthase using different CoA starters and elongation units. Putative methyl-branched and ethyl-branched FAs are highlighted in light gray and gray, respectively; ‘odd' and ‘even' qualify the total number of carbons in the FA. The asterisk indicates that the incorporation of a single methylmalonyl-CoA can also lead to the formation of ‘anteiso' FAs, which are also produced by extension of 2-methylbutyryl-CoA. (**c**) FAMEs derived from L929 adipocytes were analyzed by GC–MS. Representative EICs of the m/z corresponding to FAs with a total carbon number of 16 (m/z 270), 17 (m/z 284) or 18 (m/z 298) are shown. Monomethyl- and dimethyl-branched FA species are indicated. (**d**–**h**) Quantitative GC–MS analysis of monomethyl-branched FAs containing 17 carbons from wild-type and ECHDC1 KO L929 adipocytes clones, as well as a KO clone transduced with a recombinant lentivirus driving expression of ECHDC1 cDNA (‘ECHDC1 cDNA') or an empty vector (‘Ctrl'). Coeluting methyl-branched FAs (represented in the upper part) were quantified together. Signals were normalized to the sum of all straight chain FAs and are presented as means ± SEM from at least three independent experiments. Asterisks indicate *P* < 0.05 in post hoc testing. AU, arbitrary units.

Using CRISPR/Cas9, we generated clonal populations of 3T3-L1 and L929 cells with biallelic inactivation of ECHDC1. We then differentiated these cell lines into adipocytes and analyzed FA profiles by GC–MS. We expected to find additional peaks corresponding to the formation of methyl- or ethyl-branched FAs, if ECHDC1 indeed serves to limit the accumulation of cytoplasmic methyl- and ethylmalonyl-CoA. Total ion chromatograms (TICs) revealed only minor changes in highly abundant lipids between wild-type and KO cell lines (Supplementary Fig. S4). Yet, we noted strong increases in low abundance peaks (Figure 3a and Supplementary Fig. S4c,d) that coelute with the methyl-branched FAs that we had observed in *in vitro* reactions of FA synthase in the presence of methylmalonyl-CoA (Figure 2d,e). Monitoring the expected m/z of saturated FAs between C14 : 0 and C20 : 0 (Figure 3c and Supplementary Fig. S5a–k), we observed that peaks preceding known straight chain FAs were indeed several-fold increased in ECHDC1 KO cells (Figure 3d–h and Supplementary Fig. S5l–p).

Several complementary approaches allowed us to assign these peaks as FAs that contain methyl-branches on different even-numbered carbons as expected by methylmalonyl-CoA incorporation in FAs (Supplementary Fig. S6 for FAs containing 17 carbons). This permitted also to conclude which methyl-branched FAs contributed to which peaks in the GC–MS analysis of FA methyl esters (Figure 3d–h, upper part).

Quantification of methyl-branched FAs showed that FAs with methyl branches on C_2_, C_4_, C_6_, C_8_, C_10_ and C_12_ were the most increased (4–5-fold) in ECHDC1 KO cells, and the most decreased when ECHDC1 was re-expressed (Figure 3d–h and Supplementary Fig. S5l–p). In fact, re-expression of ECHDC1 in KO cells reduced their abundance below wild-type cells, suggesting that physiological ECHDC1 expression levels limit but do not completely prevent the formation of methyl-branched FAs.

In contrast, the 17-carbon FA containing a penultimate methyl group (the so-called iso- form) was largely unaffected by ECHDC1 inactivation or complementation, consistent with the fact that its synthesis results not from the incorporation of methylmalonyl-CoA, but from the use of a 3-methybutyryl-CoA unit derived from leucine to start FA synthesis (Figure 3b,g and Supplementary Fig. S5o). The 17-carbon FA containing an antepenultimate methyl group (the so-called anteiso- form) had an intermediary behavior as a result of the fact that it derives either from the incorporation of methylmalonyl-CoA during elongation or by using a 2-methylbutyryl-CoA derived from isoleucine to start FA synthesis (Figure 3b,h and Supplementary Fig. S5p).

Interestingly, the peak corresponding to 2-methyl-branched FAs was much higher in 3T3-L1 cells compared with L929 cells (see Figure 3c,d and Supplementary Fig. S5h,l), while the profile for the other methyl-branched FAs was similar. This suggests that the introduction of the 2-methyl branch is largely due to another enzyme than FA synthase, most likely an elongase.

Of note, KO cells showed increases in several peaks that eluted earlier than monomethyl-branched FAs (Figure 3c and Supplementary Fig. S5g–k), which based on their elution times (compared with Figure 2d and Supplementary Fig. S1b–d) are most likely dimethyl-branched. Interestingly, when we searched for the presence of unsaturated methyl-branched FAs with 14 to 18 carbons, we did not find any peak with the predicted m/z values (i.e. 240, 254, 268, 282 and 296) to be increased in ECHDC1 deficient cells compared with control cells. Similar to *in vitro* FA synthase reactions, the sensitivity of our GC–MS setup was not suffice to reveal evidence for the presence of ethyl-branched FAs.

Taken together, ECHDC1 loss leads to the accumulation of FAs carrying methyl-groups on all even-numbered carbons. The absence of unsaturated forms of these FAs might indicate that desaturases do not act efficiently on these FAs.

### ECHDC1 knockout cells accumulate ethylmalonyl-CoA but increases in cytoplasmic methylmalonyl-CoA might be hidden

**Figure 4.**
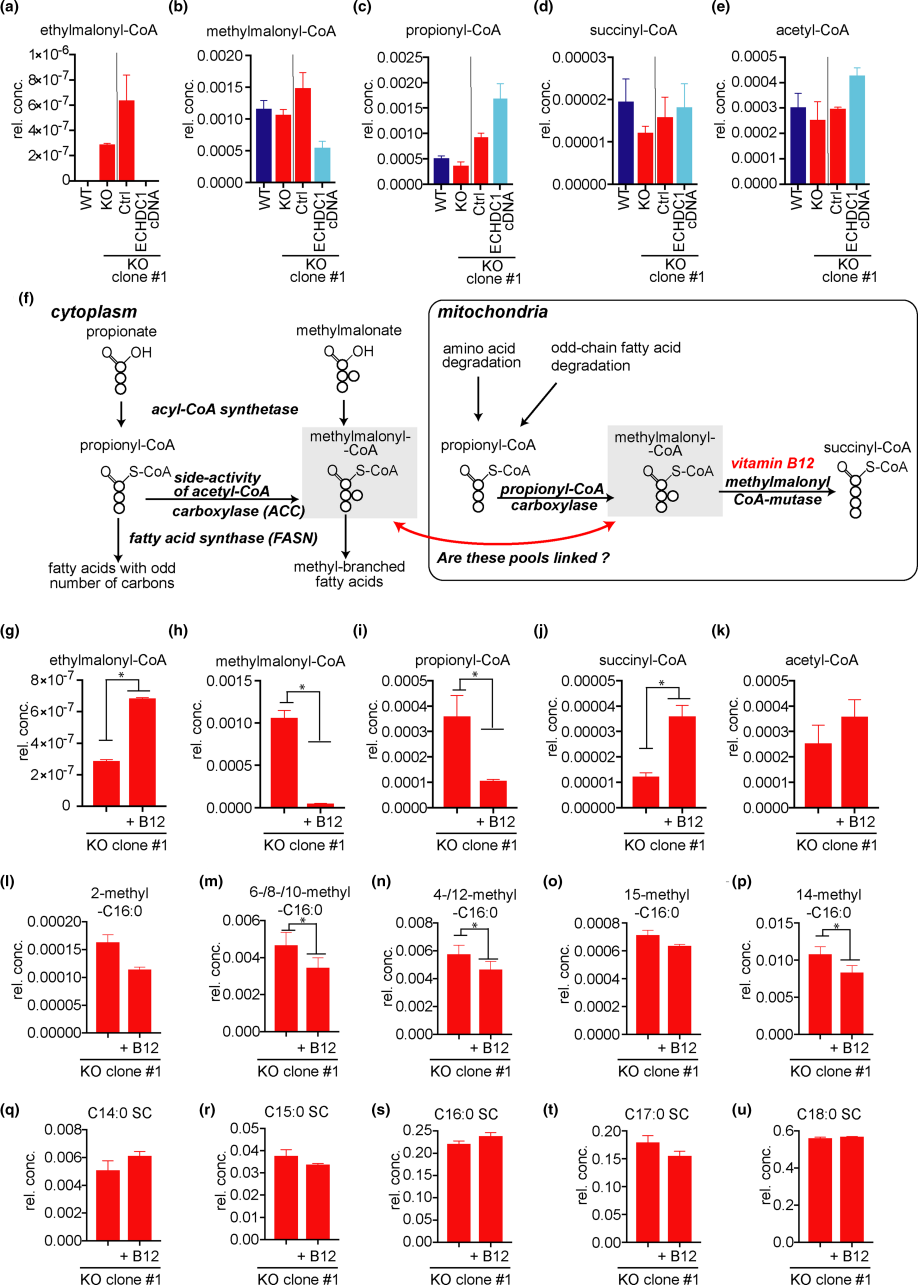
Effect of ECHDC1 deficiency on different CoA pools and effect of Vitamin B12 supplementation in ECHDC1 deficient cells. (**a**–**e**) Ethylmalonyl-CoA (**a**), methylmalonyl-CoA (**b**), propionyl-CoA (**c**), succinyl-CoA (**d**) and acetyl-CoA (**e**) were measured by LC–MS in wild-type (WT), ECHDC1 knockout (KO), as well as in a KO clone transduced with a recombinant lentivirus driving expression of ECHDC1 cDNA (‘ECHDC1 cDNA’) or an empty vector (‘Ctrl'). The vertical bar separates experiments that were performed at different points in time. (**f**) Scheme representing the formation or degradation of cytoplasmic and mitochondrial pools of methylmalonyl-CoA. Mitochondrial methylmalonyl-CoA mutase is a vitamin B12-dependent enzyme. Thus, when vitamin B12 levels are insufficient, mitochondrial methylmalonyl-CoA and propionyl-CoA are expected to increase. (**g**–**k**) Ethylmalonyl-CoA (**g**), methylmalonyl-CoA (**h**), propionyl-CoA (**i**), succinyl-CoA (**j**) and acetyl-CoA (**k**) were measured by LC–MS in ECHDC1 KO adipocytes cultured in the presence or absence of 2.5 µM supplementary vitamin B12. (**l**–**u**) monomethyl-branched FAs containing 17 carbons (**i**–**p**) and saturated straight chain (SC) FAs with 14 to 18 carbons (**q**–**u**) were assessed by GC–MS in ECHDC1 KO adipocytes in the presence or absence of 2.5 µM supplementary vitamin B12 (**q**–**u**). rel. conc. = concentration relative to total ion current for CoA esters and relative to the sum of SC fatty acids for FAs.

ECHDC1 decarboxylates ethylmalonyl-CoA and methylmalonyl-CoA [21]. To assess whether these compounds accumulate in ECHDC1 KO cells, we determined the levels of different CoA esters in cells. Ethylmalonyl-CoA was strongly increased in ECHDC1-deficient adipocytes, being undetectable in wild-type cells, while methylmalonyl-CoA levels were not significantly changed (Figure 4a,b). This is likely due to the fact that the vast majority of cellular methylmalonyl-CoA is localized in mitochondria [44]. Thus, increases in the small cytoplasmic pool of methylmalonyl-CoA might be masked. Overexpression of ECHDC1 in KO clones caused a decrease in methylmalonyl-CoA levels and an increase in propionyl-CoA, which, however, did not reach statistical significance in our statistical tests (Figure 4b,c). If confirmed, these changes are consistent with mass spectrometry data [45] indicating that ECHDC1 might partially colocalize in mitochondria. No significant change was observed in succinyl-CoA and acetyl-CoA concentrations (Figure 4d,e).

### Vitamin B12 supplementation strongly reduces methylmalonyl-CoA but barely affects methyl-branched fatty acid synthesis

Methylmalonyl-CoA can be generated in the cytoplasm by a side activity of ACC on propionyl-CoA [7] (Figs. 1 and 4f). Yet, the vast majority is produced in mitochondria by propionyl-CoA carboxylase, which uses propionyl-CoA mainly derived from the degradation of several amino acids and odd-chain FAs (Figure 4f). The resulting mitochondrial methylmalonyl-CoA can be converted by methylmalonyl-CoA mutase to succinyl-CoA in a vitamin B12-dependent reaction. Data obtained recently by different groups [46,47] indicate that cultured 3T3-L1 adipocytes may have insufficient amounts of vitamin B12. This would be expected to slow down the activity of methylmalonyl-CoA mutase and propionyl-CoA metabolism. In our LC–MS analyses, we could not distinguish between cytoplasmic and mitochondrial pools of methylmalonyl-CoA. Thus, non-physiologically high mitochondrial methylmalonyl-CoA concentrations might have masked the accumulation of cytoplasmic methylmalonyl-CoA in ECHDC1 KO cells.

Furthermore, the inability to metabolize propionyl-CoA and methylmalonyl-CoA in mitochondria might eventually lead to an unphysiological increase in the cytoplasmic pools of these metabolites. This would favor the formation of straight chain FAs with odd number of carbons and should also favor the formation of methyl-branched FAs synthesized by the incorporation of methylmalonyl-CoA (i.e. harboring the branches on even-numbered carbons) (Figure 4f). Consistent with this reflection, monomethyl-branched FAs containing methyl-branches on even carbons along with an accumulation of odd-chain FAs have been reported in the nervous system of patients suffering of impaired metabolism of vitamin B12 [48,49]. In addition, very recently Wallace and colleagues reported that 14-methyl-C17 : 0, an ‘anteiso' monomethyl-branched FA, was increased in 3T3-L1 adipocytes in the absence of vitamin B12 [2].

To test whether insufficient vitamin B12 levels contributed to the formation of methyl-branched FAs in our experimental culture conditions, we added vitamin B12 to differentiating L929 adipocytes cells. This resulted in an ∼20-fold decrease in the methylmalonyl-CoA pool and a more modest (∼4-fold) decrease in the propionyl-CoA pool (Figure 4g–k). Despite this strong reduction in cellular methylmalonyl-CoA concentration, the amount of methyl-branched FAs and odd carbon number FAs was only barely affected in L929 cells (Figure 4l–u). Of note, even under these conditions, no increase in cellular methylmalonyl-CoA was observed between wild-type and ECHDC1 KO cells (not shown). These observations indicate that the cytoplasmic pool of methylmalonyl-CoA is very small and not (or barely) connected to the large mitochondrial pool (Figure 4f). This supports our hypothesis that a side-reaction of a cytoplasmic enzyme, most likely ACC, is responsible for the formation of the cytoplasmic pool of methylmalonyl-CoA.

### Isotopic tracer studies confirm the source of the methyl-branches

**Figure 5.**
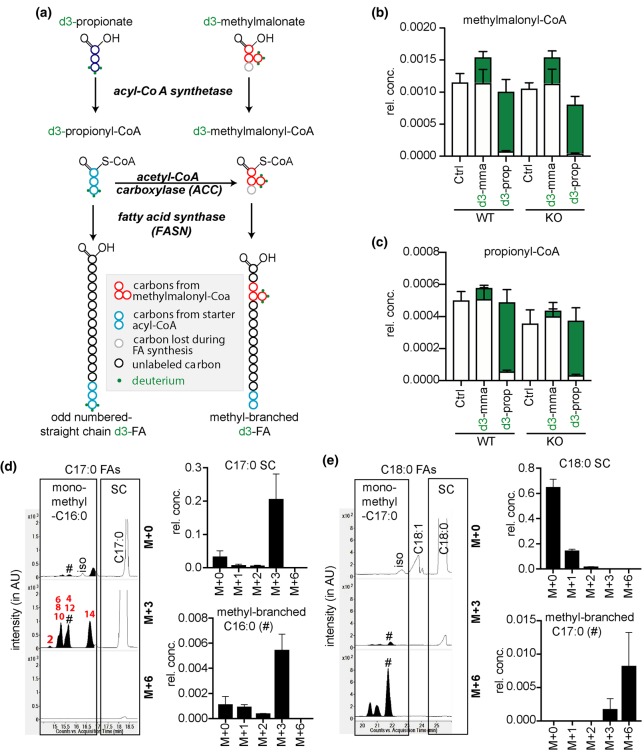
Isotopic tracer studies confirm the origin of the methyl-branches. (**a**) Expected fate of d3-propionate or d3-methylmalonate in fatty acid (FA) metabolism. (**b** and **c**) M + 0 or M + 3 methylmalonyl-CoA (**b**) and propionyl-CoA (**c**) levels were measured by LC–MS in ECHDC1 wild-type (WT) and knockout (KO) L929 cell lines treated or not (Ctrl) with 2 mM d3-methylmalonic acid (d3-mma) or d3-propionic acid (d3-prop). M + 0 and M + 3 isotopologues are presented in white and in green, respectively. Values were normalized to the total ion current and are presented as means ± SEM for three independent experiments. (**d**) GC–MS analysis of fatty acid methyl esters (FAMEs) from ECHDC1 KO L929 adipocytes fed with d3-propionate. On the left, representative EIC monitoring the molecular ion for the M + 0 C17 : 0 FAMEs (m/z 284) and the corresponding M + 3 (m/z 287) and M + 6 (m/z 290) ions. On the right, plot of the relative abundance of different isotopologues for straight chain and the indicated (indicated by # in the chromatograms) methyl-branched FAs. (**e**) Similar data as represented in (**d**), for FAs with 18 carbons (m/z 298, 301 and 304 for M + 0, M + 3 and M + 6 ions, respectively). Values are presented as means ± SEM for three independent experiments. SC, straight chain, rel. conc. = concentration relative to total ion current for CoA esters and relative to the sum of SC fatty acids for FAs; AU, arbitrary units.

To confirm that the monomethyl-branched FAs are synthesized by incorporation of methylmalonyl-CoA, we fed L929 cells with d3-methylmalonate or d3-propionate. The goal was to trace deuterium incorporation into methylmalonyl-CoA and FAs. All three deuterium atoms can only be incorporated into FAs when propionyl-CoA is used to start FA synthesis or when the usage of methylmalonyl-CoA during chain elongation introduces a methyl branch (Figure 5a).

In a first step, we measured the concentrations of deuterated CoA species (Figure 5b,c). We found that d3-propionate is a better precursor for the formation of d3-methylmalonyl-CoA and d3-propionyl-CoA than d3-methylmalonate. Addition of 2 mM d3-propionate led to a replacement of ≈95% of the unlabeled methylmalonyl-CoA by an almost equivalent amount of d3-methylmalonyl-CoA (Figure 5b).

In the presence of d3-propionate, both the straight chain C17 : 0 and the monomethyl-branched C16 : 0 FAs mostly showed a M + 3 labeling (Figure 5d). This is expected since one propionyl-CoA, as such for the straight chain C17 : 0 and as methylmalonyl-CoA for monomethyl-branched C16 : 0, is needed to synthesize these FAs. For monomethyl-branched C17 : 0 FAs, the M + 6 labeling is the most intense (Figure 5e), in agreement with the fact that two propionyl-CoA molecules are needed for their synthesis: as a starting unit, and as methylmalonyl-CoA in one of the extension steps. As expected, straight chain C18 : 0 was not labeled.

### Methyl-branched fatty acids are incorporated into various lipid species in mammalian adipocytes

**Figure 6.**
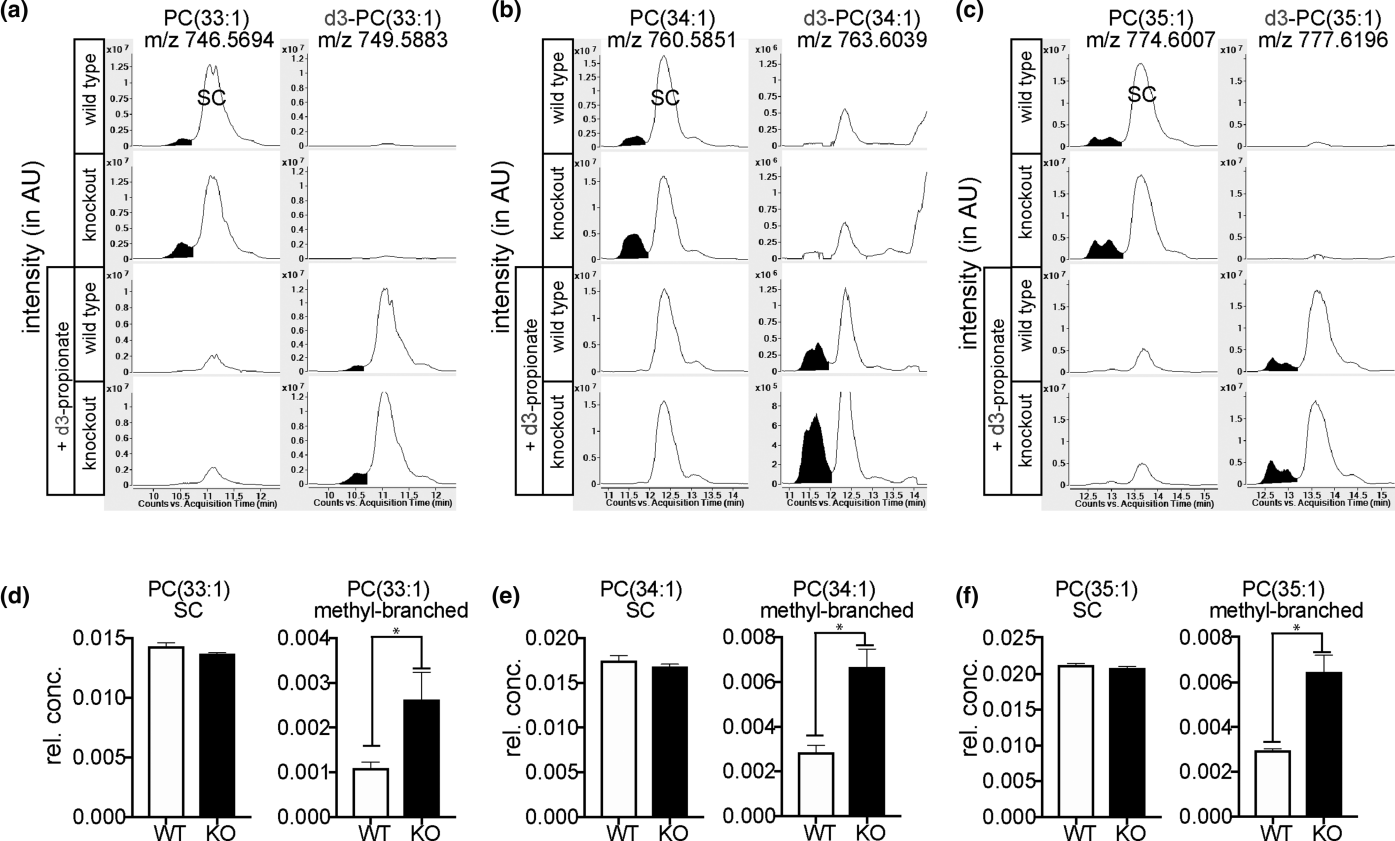
Methyl-branched fatty acids are incorporated in PC. (**a**–**c**) Representative EICs from lipidomic LC–MS analysis, using ESI in the positive mode, of PC(33 : 1), PC(34 : 1) and PC(35 : 1) in wild-type (WT) or ECHDC1 knockout (KO) L929 adipocytes incubated without (upper tracings) or with (lower tracings) 2 mM d3-propionate. PC species containing straight chain (SC) FAs are indicated and methyl-branched FAs are colored in black. (**d**–**f**) Quantitative analysis of the same data representing means ± SEM for three independent experiments in triplicates. Rel. conc., relative concentration (to total ion current). Related data on other lipid species are shown in Supplementary Figure S7. AU, arbitrary units, rel.conc. = concentration relative to total ion current.

While the majority of FAs in adipocytes are esterified in triglycerides, we were interested to see whether methyl-branched FAs were also incorporated in other lipid species. To this end, we analyzed the organic phase of wild-type and ECHDC1 KO cells by LC–MS and focused on membrane lipids (phosphatidylcholine (PC), phosphatidylethanolamine and sphingomyelin). We expected that branched FA-containing membrane lipids would elute before their counterparts that only contained straight chains. As exemplified for some PC species (Figure 6), we indeed observed that several overlapping peaks preceded a major peak with the same m/z. The size of these small peaks increased by ∼2-fold in ECHDC1 KO cells (Figure 6). Furthermore, when we performed the same experiment in the presence of d3-propionate, they were replaced by peaks with a M + 3 increment at the same elution time (Figure 6a–c, lower panel). Thus, the most likely explanation is that the labeled small peak(s) correspond(s) to PC species that contain methyl-branches, whereas the labeled big peak corresponds to PC species that contain FAs with an odd carbon number. Similar analyses revealed incorporation of putative methyl-branched FAs in phosphatidylethanolamine (PE) and sphingomyelin (SM) species (Supplementary Fig. S7).

### Evidence for the synthesis of ethyl-branched fatty acids in cultured adipocytes

**Figure 7.**
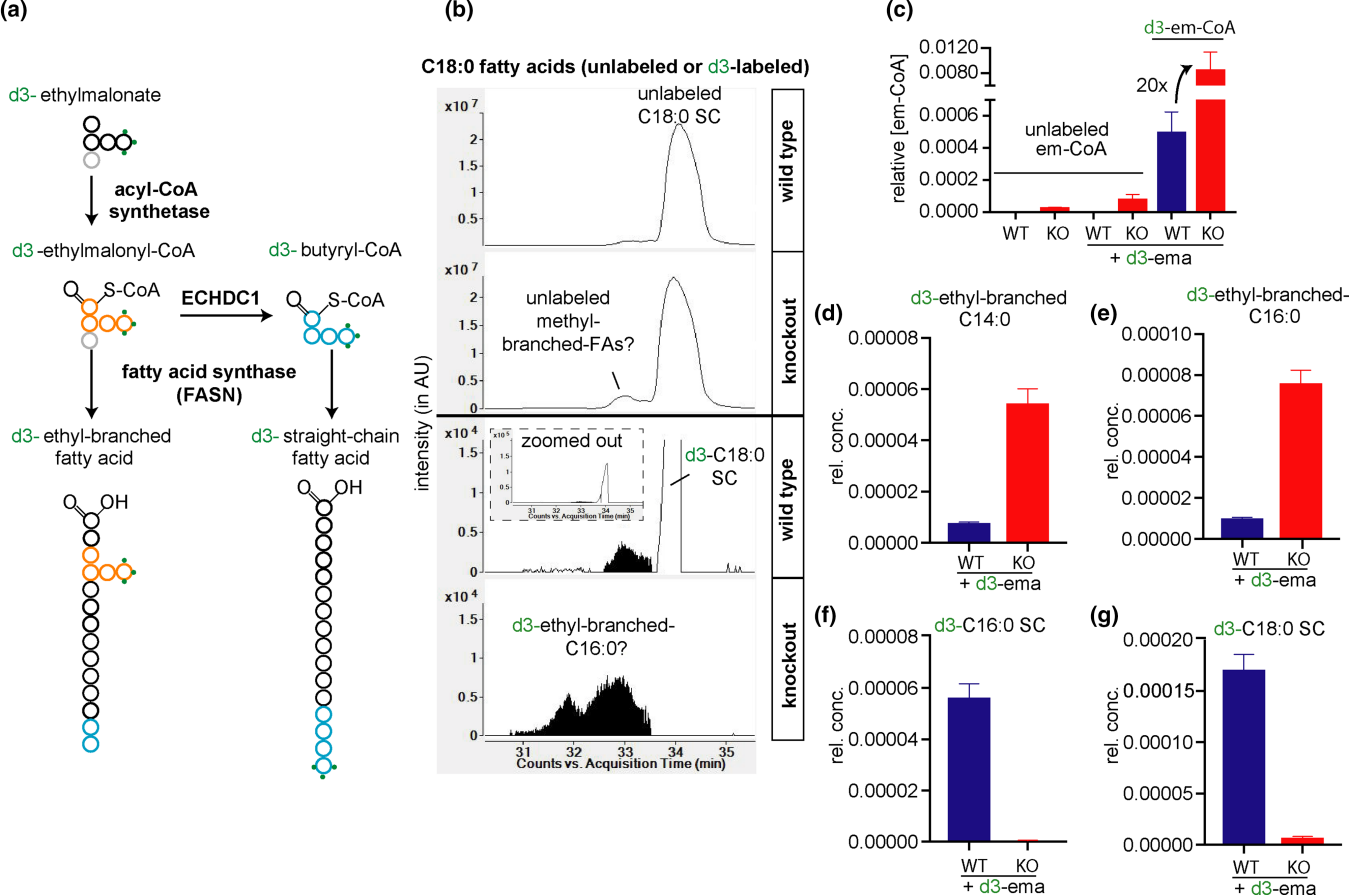
Isotopic tracer studies reveal that ethyl-branched fatty acids almost exclusively accumulate in ECHDC1 knockout cells. (**a**) Expected fate of d3-ethylmalonate in FA synthesis. (**b**) Representative EIC in LC–MS showing unlabeled (m/z 283.26425) and labeled (m/z 286.28308) free FAs with 18 carbons, released from L929 adipocytes extracts. SC, straight chain. Representative EIC corresponding to unlabeled C18 : 0 (m/z 283.26425) (upper tracings) and d3-labeled C18 : 0 (m/z 286.28308) (lower tracings) for wild-type (WT) and ECHDC1 knockout (KO) cells, as indicated. (**c**) Quantitative analysis of unlabeled or labeled ethylmalonyl-CoA in L929 adipocytes upon treatment with d3-ethylmalonate. (**d**) Quantitative analysis of putative labeled ethyl-branched C14 : 0 (16 carbons in total) in L929 wild-type and ECHDC1 KO adipocytes. (**e**) Similar figure as d, for putative labeled ethyl-branched C16 : 0 (18 carbons in total). (**f**) Quantitative analysis of labeled straight chain C16 : 0 in L929 wild-type and ECHDC1 KO adipocytes. (**g**) Similar figure as **f**, for labeled straight chain C18 : 0. AU, arbitrary units, rel.conc. = concentration relative to total ion current.

To investigate the formation of ethyl-branched FAs in ECHDC1 KO cells, we performed LC–MS analyses. We incubated the cells with d3-ethylmalonate to label and inflate the pool of intracellular ethylmalonyl-CoA (Figure 7a). As expected, this led to a marked (100-fold) increase in the ethylmalonyl-CoA pool in the ECHDC1-deficient cells, which reached a concentration more than 10-fold higher than in wild-type cells (Figure 7c).

Consistent with our *in vitro* observation that FA synthase is slowly using ethylmalonyl-CoA, we detected the formation of labeled putative ethyl-branched FAs when we fed the cells with d3-ethylmalonate. Knocking out of ECHDC1 strongly increased the accumulation of these metabolites, which have similar retention time as ethyl-branched FAs formed *in vitro* by FA synthase (Figure 2h). The only FAs that we found to be modified in this way contained 16 and 18 carbons (Figure 7b lower part, 7d,e). Consistent with the expected labeling of butyryl-CoA in the presence of ECHDC1 and with its incorporation in FAs (Figure 7a), wild-type cells synthesized labeled straight chain FAs (coeluting with unlabeled straight chain FAs), while this was not the case for the ECHDC1-deficient cells (Figure 7b,f,g)

Overall, we conclude that ECHDC1 KO cells can produce ethyl-branched FAs. Yet, the formation of these FAs is prevented in normal cells due to the action of ECHDC1 and due to the fact that ethylmalonyl-CoA is a poor substrate for the elongation steps by FA synthase.

## Discussion

### Fatty acid synthase can make methyl- and ethyl-branched fatty acids

We confirmed that FA synthase is able to synthesize FAs with methyl-branches and that methylmalonyl-CoA is a poorer substrate than malonyl-CoA. Analysis of the reaction products revealed that FA synthase can introduce several methyl-branches and even generate polymethylated-FAs using only methylmalonyl-CoA for elongation in the absence of malonyl-CoA.

We also showed that FA synthase is able to use ethylmalonyl-CoA to form ethyl-branched FAs and that ethylmalonyl-CoA is a much poorer substrate than methylmalonyl-CoA. In the absence of standards, the quantitative analysis of the resulting ethyl-branched FAs was difficult. Furthermore, due to insufficient sensitivity of our GC–MS setup, we needed to analyze ethyl-branched FAs by LC–MS. Based on the known sequence of reactions in FA synthase, ethyl-branches should be attached to even-numbered carbon atoms. Yet, the softer ionization and fragmentation techniques used in LC–MS did not allow us to directly confirm this.

Overall, our analyses reveal that both methylmalonyl-CoA and ethylmalonyl-CoA can be used by FA synthase, albeit with reduced efficiency in comparison with malonyl-CoA. It is likely that some elongases are also able to use methyl- or ethylmalonyl-CoA instead of malonyl-CoA, as supported by the finding of more abundant 2-methyl-branched FAs in 3T3-L1 cells than in L929 cells. This suggests that cytoplasmic concentrations of (m)ethylmalonyl-CoA most likely determine to what extent branched FAs are being formed in cells. Due to its ability to decarboxylate (m)ethylmalonyl-CoA, ECHDC1 is expected to be a major determining factor for branched FA synthesis.

### Methyl-branched fatty acids are present in normal cells but their formation is limited by ECHDC1

**Figure 8.**
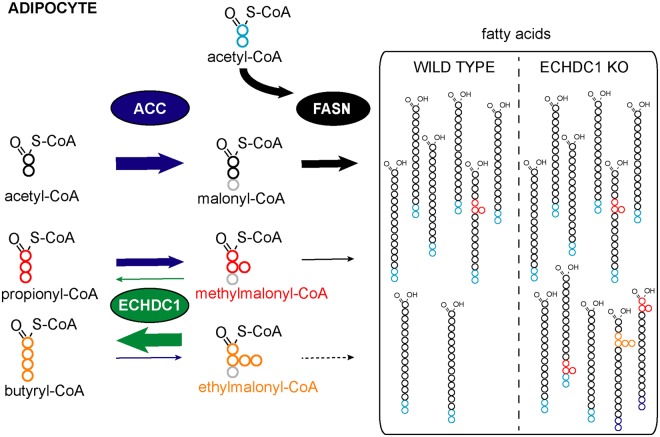
ECHDC1 limits the synthesis of methyl-branched fatty acids and prevents the synthesis of ethyl-branched fatty acids in adipocytes. ACC, acetyl-CoA carboxylase; FASN, fatty acid synthase; ECHDC1, (m)ethylmalonyl-CoA decarboxylase; KO, knockout. The size of the arrows reflects the catalytic efficiency of the enzymes. Carbons in gray are not incorporated in fatty acids.

When we inactivated ECHDC1 in two adipocyte cell culture models, we observed up to 5-fold increases in methyl-branched FAs. Using synthetic standards and the analysis of the fragmentation pattern of two kinds of FA derivatives (Supplementary Fig. S6), we found that this increase concerned FAs that carried methyl-branches on any of the even-numbered carbons. This unequivocally establishes that ECHDC1 limits the formation of methyl-branched FAs in cells (Figure 8).

Remarkably, wild-type cells also had these branched FAs, but overexpression of ECHDC1 (Figure 3d–h, lower part) reduced these methyl-branched FAs to almost undetectable levels. This indicates that changes in ECHDC1 abundance or activity are likely to result in reciprocal changes of the abundance of methyl-branched FAs.

To avoid any misinterpretation of our results, we highlight that these methyl-branched FAs are fundamentally different from ‘anteiso-’ and ‘iso-’ forms of methyl-branched FAs, which carry methyl-groups on the penultimate and antepenultimate carbon atoms. These FAs have received quite recently considerable interest (Wallace et al. [2]) and can be synthesized when FA synthase uses break-down products of branched-chain amino acids to start FA synthesis (see Figure 3b). To the best of our knowledge, very little is known about the physiological role of FAs containing methyl-groups on even-numbered carbons. Tuning of ECHDC1 expression or activity might allow us to modulate the abundance of these FAs and investigate their function.

### Why do we need to limit the formation of methyl-branched fatty acids?

From a metabolic standpoint, it is not clear why cells would need to limit the synthesis of methyl-branched FAs since mammalian cells have efficient metabolic pathways to metabolize methyl-branched FAs. Peroxisomal disorders with deficiencies in these pathways (peroxisomal alpha- or beta-oxidation) lead to severe neurological disease (Van Veldhoven [17]; Wanders [18]) due to the accumulation of nonmetabolizable methyl-branched FAs in tissues.

ECHDC1 deficiency *in vivo* should lead to an increase in methyl-branched FAs. Overall, our organism should be able to prevent excessive accumulation of these FAs, since their degradation is not expected to be changed. Yet, expression levels of enzymes involved in the degradation of methyl-branched FAs vary between tissues. Therefore, we cannot exclude that some tissues would be more efficient than others in limiting the accumulation of methyl-branched FAs.

Methyl-branched FAs seem to be integrated not only in triglycerides, but also in membrane lipids. This might be expected to alter membrane physiology. *In vitro* experiments document that methyl-branched FAs can reduce lipid bilayer thickness and membrane fluidity [50] and the incorporation of methyl-branched FAs in membrane lipids has been reported to modulate processes that depend on membrane fluidity such as respiratory activity in bacteria [6]. Thus, it is conceivable that modulation of methyl-branched FA levels might finetune similar processes in mammals.

Beyond the presence of the methyl-branches, membrane behavior might also be altered due to the changes in FA unsaturation. In our cell line models, we were unable to detect any methyl-branched FAs on even carbons with a double bond, suggesting interference with the desaturase activities. In some tissues, it might be physiologically relevant to synthesize FAs that are branched and strictly saturated. To conclude, regulation of ECHDC1 activity or expression might be a way to modulate the formation of cytosolic methylmalonyl-CoA and methyl-branched FAs.

### The cytoplasmic and mitochondrial methylmalonyl-CoA pools are independent

Under standard culture conditions, our adipocyte models have access to limited amounts of vitamin B12 (Crown et al. [46]; Green et al. [47]; Wallace et al. [2]). Remarkably, when we supplemented cells with this vitamin, total cellular methylmalonyl-CoA levels were 20-fold reduced. In contrast, synthesis of methyl-branched FAs was barely affected. This indicates that cytosolic methylmalonyl-CoA, which is used for the synthesis of methyl-branched FAs, originates from the carboxylation of propionyl-CoA by ACC (Waite and Wakil [7]) rather than from the transfer of mitochondrial methylmalonyl-CoA to the cytosol.

### The combination of ECHDC1 activity and the substrate specificity of fatty acid synthase completely avoid ethyl-branched fatty acid synthesis

ECHDC1 KO cells show clearly detectable ethylmalonyl-CoA levels, whereas this metabolite is undetectable in wild-type cells. This indicates that ECHDC1 very efficiently prevents the accumulation of this metabolite. Our initial *in vitro* experiments had revealed that ethylmalonyl-CoA is a much poorer substrate for FA synthase than methylmalonyl-CoA. Thus, the formation of ethyl-branched FAs is first prevented by ECHDC1, which completely eliminates ethylmalonyl-CoA, and second, by the poor efficiency of FA synthase to use ethylmalonyl-CoA for chain elongation (Figure 8).

In contrast with methyl-branched FAs, some data indicate that mammals are unable to metabolize ethyl-branched FAs. For example, humans are unable to completely degrade 2-ethylhexanoic acid, a component of plasticizers, since its major metabolite found in urine is 2-ethyl-3-ketohexanoic acid [51]. It is conceivable that the degradation of longer ethyl-branched FAs might lead to compounds that are not as easily excreted as 2-ethyl-3-ketohexanoic acid. This might lead to a diverse range of acute toxicity or progressive accumulation in intracellular deposits. Whether this ever becomes a clinically relevant problem is unknown and, until now, a deficiency in ECHDC1 has never been described in humans.

By metabolizing ethylmalonyl-CoA, a compound with no known function in vertebrates, ECHDC1 seems to play a typical metabolite repair role. Many metabolite repair enzymes have been described in recent years. They serve to destroy or recycle metabolites that are formed by enzyme side activities or through spontaneous reactions. Their importance is stressed by the rapidly growing number of enzymes playing this role and by the fact that a deficiency in several of them, such as L-2-hydroxyglutarate dehydrogenase [52], NADHX epimerase [53], NADHX dehydratase [54], and G6PC3 [39] leads to diseases in humans. ECHDC1 is the first such repair enzyme to be described in the context of lipid metabolism. Further research is needed to know if its deficiency leads to a human disease and if other repair enzymes are involved in lipid metabolism.

## Abbreviations

ACC: acetyl-CoA carboxylase
AU: Arbitrary unit
ECHDC1: ethylmalonyl-CoA decarboxylase
EIC: extracted ion chromatogram
ESI: electrospray ionization
FA: fatty acid
FAME: fatty acid methyl ester
FASN: fatty acid synthase
GC–MS: gas chromatography–mass spectrometry
LC–MS: liquid chromatography–mass spectrometry
PC: phosphatidylcholine
SC: straight chain
SIM: selected-ion monitoring

## References

[1] Horning M.G., Martin D.B., Karmen A. and Vagelos P.R. (1961) Fatty acid synthesis in adipose tissue. II. Enzymatic synthesis of branched chain and odd-numbered fatty acids. J. Biol. Chem. 236, 669–672 PMID:13715907

[2] Wallace M., Green C.R., Roberts L.S., Lee Y.M., McCarville J.L., Sanchez-Gurmaches J. et al. (2018) Enzyme promiscuity drives branched-chain fatty acid synthesis in adipose tissues. Nat. Chem. Biol. 14, 1021–1031 10.1038/s41589-018-0132-2 30327559 PMC6245668

[3] Kaneda T. (1991) Iso- and anteiso-fatty acids in bacteria: biosynthesis, function, and taxonomic significance. Microbiol. Rev. 55, 288–302 PMID:1886522 10.1128/mr.55.2.288-302.1991 PMC372815

[4] Kniazeva M., Crawford Q.T., Seiber M., Wang C.Y. and Han M. (2004) Monomethyl branched-chain fatty acids play an essential role in *Caenorhabditis elegans* development. PLoS Biol. 2, E257 10.1371/journal.pbio.0020257 15340492 PMC514883

[5] Oku H., Yagi N., Nagata J. and Chinen I. (1994) Precursor role of branched-chain amino acids in the biosynthesis of iso and anteiso fatty acids in rat skin. Biochim. Biophys. Acta 1214, 279–287 10.1016/0005-2760(94)90074-4 7918610

[6] Budin I., de Rond T., Chen Y., Chan L.J.G., Petzold C.J. and Keasling J.D. (2018) Viscous control of cellular respiration by membrane lipid composition. Science 362, 1186–1189 10.1126/science.aat7925 30361388

[7] Waite M. and Wakil S.J. (1962) Studies on the mechanism of fatty acid synthesis. XII. Acetyl coenzyme A carboxylase. J. Biol. Chem. 237, 2750–2757 PMID:14004432

[8] Cardinale G.J., Carty T.J. and Abeles R.H. (1970) Effect of methylmalonyl coenzyme A, a metabolite which accumulates in vitamin B 12 deficiency, on fatty acid synthesis. J. Biol. Chem. 245, 3771–3775 PMID:5492945

[9] Buckner J.S., Kolattukudy P.E. and Rogers L. (1978) Synthesis of multimethyl-branched fatty acids by avian and mammalian fatty acid synthetase and its regulation by malonyl-CoA decarboxylase in the uropygial gland. Arch. Biochem. Biophys. 186, 152–163 10.1016/0003-9861(78)90474-5 629531

[10] Kolattukudy P.E., Rogers L.M. and Balapangu A. (1987) Synthesis of methyl-branched fatty acids from methylmalonyl-CoA by fatty acid synthase from both the liver and the harderian gland of the Guinea pig. Arch. Biochem. Biophys. 255, 205–209 10.1016/0003-9861(87)90312-2 3592662

[11] Murray K. (1962) Studies in waxes. XXI. The branched-chain acids of the preen gland wax of the goose. Aus. J. Chem. 15, 510–520 10.1071/CH9620510

[12] Smith A. and Duncan W.R. (1979) Characterization of branched-chain fatty acids from fallow deer perinephric triacylglycerols by gas chromatography-mass spectrometry. Lipids 14, 350–355 10.1007/BF02533418 440025

[13] Seyama Y., Ohashi K., Imamura T., Kasama T. and Otsuka H. (1983) Branched chain fatty acids in phospholipids of Guinea pig Harderian gland. J. Biochem. 94, 1231–1239 10.1093/oxfordjournals.jbchem.a134468 6654855

[14] Alves S.P., Bessa R.J., Quaresma M.A., Kilminster T., Scanlon T., Oldham C. et al. (2013) Does the fat tailed Damara ovine breed have a distinct lipid metabolism leading to a high concentration of branched chain fatty acids in tissues? PLoS ONE 8, e77313 10.1371/journal.pone.0077313 24204803 PMC3800059

[15] Buckner J.S. and Kolattukudy P.E. (1975) Lipid biosynthesis in the sebaceous glands: synthesis of multibranched fatty acids from methylmalonyl-coenzyme A in cell-free preparations from the uropygial gland of goose. Biochemistry 14, 1774–1782 10.1021/bi00679a033 235967

[16] Kim Y.S. and Kolattukudy P.E. (1978) Malonyl-CoA decarboxylase from the uropygial gland of waterfowl: purification, properties, immunological comparison, and role in regulating the synthesis of multimethyl-branched fatty acids. Arch. Biochem. Biophys. 190, 585–597 10.1016/0003-9861(78)90314-4 102255

[17] Van Veldhoven P.P. (2010) Biochemistry and genetics of inherited disorders of peroxisomal fatty acid metabolism. J. Lipid Res. 51, 2863–2895 10.1194/jlr.R005959 20558530 PMC2936746

[18] Wanders R.J. (2014) Metabolic functions of peroxisomes in health and disease. Biochimie 98, 36–44 10.1016/j.biochi.2013.08.022 24012550

[19] Jacob J. and Poltz J. (1974) Chemical composition of uropygial gland secretions of owls. J. Lipid Res. 15, 243–248 PMID:4827914

[20] Hillbrick G., Tucker D. and Smith G. (1995) The lipid composition of cashmere goat fleece. Aus. J. Agric. Res. 46, 1259–1271 10.1071/AR9951259

[21] Linster C.L., Noel G., Stroobant V., Vertommen D., Vincent M.F., Bommer G.T. et al. (2011) Ethylmalonyl-CoA decarboxylase, a new enzyme involved in metabolite proofreading. J. Biol. Chem. 286, 42992–43003 10.1074/jbc.M111.281527 22016388 PMC3234807

[22] Linster C.L., Van Schaftingen E. and Hanson A.D. (2013) Metabolite damage and its repair or pre-emption. Nat. Chem. Biol. 9, 72–80 10.1038/nchembio.1141 23334546

[23] Peracchi A. (2018) The limits of enzyme specificity and the evolution of metabolism. Trends Biochem. Sci. 43, 984–996 10.1016/j.tibs.2018.09.015 30472990

[24] Bommer G.T., Schaftingen E.V. and Veiga-da-Cunha M. (2019) Metabolite repair enzymes control metabolic damage in glycolysis. Trends Biochem. Sci. Accepted, 10.1016/j.tibs.2019.07.004 31473074

[25] Hemati N., Ross S.E., Erickson R.L., Groblewski G.E. and MacDougald O.A. (1997) Signaling pathways through which insulin regulates CCAAT/enhancer binding protein alpha (C/EBPα) phosphorylation and gene expression in 3T3-L1 adipocytes. Correlation with GLUT4 gene expression. J. Biol. Chem. 272, 25913–25919 10.1074/jbc.272.41.25913 9325324

[26] Cong L., Ran F.A., Cox D., Lin S., Barretto R., Habib N. et al. (2013) Multiplex genome engineering using CRISPR/Cas systems. Science 339, 819–823 10.1126/science.1231143 23287718 PMC3795411

[27] Ran F.A., Hsu P.D., Wright J., Agarwala V., Scott D.A. and Zhang F. (2013) Genome engineering using the CRISPR-Cas9 system. Nat. Protoc. 8, 2281–2308 10.1038/nprot.2013.143 24157548 PMC3969860

[28] Sanjana N.E., Shalem O. and Zhang F. (2014) Improved vectors and genome-wide libraries for CRISPR screening. Nat. Methods 11, 783–784 10.1038/nmeth.3047 25075903 PMC4486245

[29] Hart T., Chandrashekhar M., Aregger M., Steinhart Z., Brown K.R., MacLeod G. et al. (2015) High-resolution CRISPR screens reveal fitness genes and genotype-specific cancer liabilities. Cell 163, 1515–1526 10.1016/j.cell.2015.11.015 26627737

[30] Lorenz M.A., Burant C.F. and Kennedy R.T. (2011) Reducing time and increasing sensitivity in sample preparation for adherent mammalian cell metabolomics. Anal. Chem. 83, 3406–3414 10.1021/ac103313x 21456517 PMC3094105

[31] Matyash V., Liebisch G., Kurzchalia T.V., Shevchenko A. and Schwudke D. (2008) Lipid extraction by methyl-tert-butyl ether for high-throughput lipidomics. J. Lipid Res. 49, 1137–1146 10.1194/jlr.D700041-JLR200 18281723 PMC2311442

[32] Bijleveld C. and Geelen M.J. (1987) Measurement of acetyl-CoA carboxylase activity in isolated hepatocytes. Biochim. Biophys. Acta 918, 274–283 10.1016/0005-2760(87)90231-1 2882781

[33] Baquet A., Gaussin V., Bollen M., Stalmans W. and Hue L. (1993) Mechanism of activation of liver acetyl-CoA carboxylase by cell swelling. Eur. J. Biochem. 217, 1083–1089 10.1111/j.1432-1033.1993.tb18340.x 7901014

[34] Aveldano M.I. and Horrocks L.A. (1983) Quantitative release of fatty acids from lipids by a simple hydrolysis procedure. J. Lipid Res. 24, 1101–1105 PMID:6631237

[35] Collard F., Baldin F., Gerin I., Bolsee J., Noel G., Graff J. et al. (2016) A conserved phosphatase destroys toxic glycolytic side products in mammals and yeast. Nat. Chem. Biol. 12, 601–607 10.1038/nchembio.2104 27294321

[36] Ichihara K. and Fukubayashi Y. (2010) Preparation of fatty acid methyl esters for gas-liquid chromatography. J. Lipid Res. 51, 635–640 10.1194/jlr.D001065 19759389 PMC2817593

[37] Destaillats F. and Angers P. (2002) One-step methodology for the synthesis of FA picolinyl esters from intact lipids. J. Amer. Oil Chem. Soc. 79, 253–256 10.1007/s11746-002-0469-7

[38] Peter D.M., Vogeli B., Cortina N.S. and Erb T.J. (2016) A chemo-enzymatic road map to the synthesis of CoA esters. Molecules 21, 517 10.3390/molecules21040517 27104508 PMC6273144

[39] Veiga-da-Cunha M., Chevalier N., Stephenne X., Defour J.P., Paczia N., Ferster A. et al. (2019) Failure to eliminate a phosphorylated glucose analog leads to neutropenia in patients with G6PT and G6PC3 deficiency. Proc. Natl Acad. Sci. U.S.A. 116, 1241–1250 10.1073/pnas.1816143116 30626647 PMC6347702

[40] Benjamini Y. and Hochberg Y. (1995) Controlling the false discovery rate: a practical and powerful approach to multiple testing. J. R. Stat. Soc. B. 57, 289–300 10.2307/2346101

[41] Apon J.M. and Nicolaides N. (1975) The determination of the position isomers of the methyl branches fatty acids methyl esters by capillary GC/MS. J. Chromatogr. Sci. 13, 467–473 10.1093/chromsci/13.10.467 1184713

[42] Lira M.C., Rosa F.D., Panelo L.C., Costas M.A. and Rubio M.F. (2018) Role of RAC3 coactivator in the adipocyte differentiation. Cell Death Discov. 4, 20 PMID:30062065 10.1038/s41420-018-0085-y PMC6062518

[43] Bennett C.N., Longo K.A., Wright W.S., Suva L.J., Lane T.F., Hankenson K.D. et al. (2005) Regulation of osteoblastogenesis and bone mass by Wnt10b. Proc. Natl Acad. Sci. U.S.A. 102, 3324–3329 10.1073/pnas.0408742102 15728361 PMC552924

[44] Frenkel E.P. and Kitchens R.L. (1975) Intracellular localization of hepatic propionyl-CoA carboxylase and methylmalonyl-CoA mutase in humans and normal and vitamin B12 deficient rats. Br. J. Haematol. 31, 501–513 10.1111/j.1365-2141.1975.tb00885.x 24458

[45] Jadot M., Boonen M., Thirion J., Wang N., Xing J., Zhao C. et al. (2017) Accounting for protein subcellular localization: a compartmental map of the rat liver proteome. Mol. Cell Proteomics 16, 194–212 10.1074/mcp.M116.064527 27923875 PMC5294208

[46] Crown S.B., Marze N. and Antoniewicz M.R. (2015) Catabolism of branched chain amino acids contributes significantly to synthesis of odd-chain and even-chain fatty acids in 3T3-L1 adipocytes. PLoS ONE 10, e0145850 10.1371/journal.pone.0145850 26710334 PMC4692509

[47] Green C.R., Wallace M., Divakaruni A.S., Phillips S.A., Murphy A.N., Ciaraldi T.P. et al. (2016) Branched-chain amino acid catabolism fuels adipocyte differentiation and lipogenesis. Nat. Chem. Biol. 12, 15–21 10.1038/nchembio.1961 26571352 PMC4684771

[48] Kishimoto Y., Williams M., Moser H.W., Hignite C. and Biermann K. (1973) Branched-chain and odd-numbered fatty acids and aldehydes in the nervous system of a patient with deranged vitamin B 12 metabolism. J. Lipid Res. 14, 69–77 PMID:4701555

[49] Ramsey R.B., Scott T. and Banik N.L. (1977) Fatty acid composition of myelin isolated from the brain of a patient with cellular deficiency of co-enzyme forms of vitamin B12. J. Neurol. Sci. 34, 221–232 10.1016/0022-510X(77)90070-3 925711

[50] Poger D., Caron B. and Mark A.E. (2014) Effect of methyl-branched fatty acids on the structure of lipid bilayers. J. Phys. Chem. B 118, 13838–13848 10.1021/jp503910r 25380125

[51] Stingel D., Feldmeier P., Richling E., Kempf M., Elss S., Labib S. et al. (2007) Urinary 2-ethyl-3-oxohexanoic acid as major metabolite of orally administered 2-ethylhexanoic acid in human. Mol. Nutr. Food Res. 51, 301–306 PMID:17309117 10.1002/mnfr.200600136

[52] Rzem R., Vincent M.F., Van Schaftingen E. and Veiga-da-Cunha M. (2007) L-2-hydroxyglutaric aciduria, a defect of metabolite repair. J. Inherit. Metab. Dis. 30, 681–689 10.1007/s10545-007-0487-0 17603759

[53] Kremer L.S., Danhauser K., Herebian D., Petkovic Ramadza D., Piekutowska-Abramczuk D., Seibt A. et al. (2016) NAXE mutations disrupt the cellular NAD(P)HX repair system and cause a lethal neurometabolic disorder of early childhood. Am. J. Hum. Genet. 99, 894–902 10.1016/j.ajhg.2016.07.018 27616477 PMC5065653

[54] Van Bergen N.J., Guo Y., Rankin J., Paczia N., Becker-Kettern J., Kremer L.S. et al. (2019) NAD(p)HX dehydratase (NAXD) deficiency: a novel neurodegenerative disorder exacerbated by febrile illnesses. Brain 142, 50–58 10.1093/brain/awy310 30576410

