# Supplementary material for: The synthesis of branched-chain fatty acids is limited by enzymatic decarboxylation of ethyl- and methylmalonyl-CoA
Source: Biochem J. 2019 Aug 30;476(16):2427–47. doi: 10.1042/BCJ20190500 (PMC6717113; doi:10.1042/BCJ20190500)
Supplement: Supplementary Figures [file BCJ-476-2427-s1.pdf]

SUPPLEMENTARY FIGURES LEGENDS

Figure S1. Fatty acid synthase synthesizes various methyl-branched fatty acids by incorporation of methylmalonyl-CoA

Same experiment as in Fig. 2. (**a**) Table representing the expected FAs according to their number of carbons and the incorporation of malonyl-CoA or methylmalonyl-CoA unit(s). (**b**)-(**d**) GC-MS analysis of the produced deuterated fatty acids (FAs) with total carbon number of 15 (m/z 259), 16 (m/z 273), and 18 (m/z 301), (**e**)-(**k**) LC-MS analysis of deuterated FAs with even numbers of carbons between 8 and 20 (m/z 146.1266, 174.1579, 202.1892, 230.2205, 258.2518, 286. 2831 and 314.3144, respectively). (**l**)-(**r**) LC-MS analysis of deuterated FAs with odd numbers of carbons between 9 and 21 (m/z 160.1423, 188.1734, 216.2049, 244.236, 272.2675, 300.2988 and 328.3301, respectively). Putative methyl-branched FAs are indicated in black. The data shown are representative of at least 3 independent experiments. AU, arbitrary units.

Figure S2. Relationship between the amount of mono- or polymethyl-fatty acids synthesized by fatty acid synthase and the concentration of methylmalonyl-CoA

(**a**) Sum of monomethyl-, dimethyl-, trimethyl- and tetramethyl-branched fatty acids (FAs) monitored by GC-MS analysis of fatty acid methylesters. (**b**) Sum of monomethyl-, dimethyl-, trimethyl-, tetramethyl and pentamethyl-branched FAs monitored by LC-MS analysis. Values are presented as means +/- SEM from three independent experiments.

Figure S3. Fatty acid synthase can synthesize ethyl-branched fatty acids by incorporation of ethylmalonyl-CoA (LC-MS)

LC-MS analysis of the fatty acids (FAs) produced after incubation of FA synthase with malonyl-CoA, d3-acetyl-CoA and the indicated concentrations of ethylmalonyl-CoA. (**a**)-(**g**) Representative EICs of m/z corresponding to deuterated fatty acids with even numbers of carbons between 8 and 20 (same m/z as Fig. S1 (e)-(k)). Putative monoethyl- and diethyl-branched FAs are indicated in black. (**h**) Relationship between the sum of monoethyl- and diethyl-branched FAs monitored by LC-MS analysis and the concentration of ethylmalonyl-CoA used in the synthesis. AU, arbitrary units.

Figure S4. Fatty acid profiles of L929 and 3T3-L1 adipocytes analyzed by GC-MS in SCAN mode

Fatty acid (FA) profiles obtained by GC-MS analysis of fatty acid methylesters (FAMEs) from L929 and 3T3-L1 cells before and after adipocyte differentiation revealed similarities and differences. In both cell lines, we observed an increase in monounsaturated FAs and an increase in odd carbon number FAs as previously described (46). The most abundant FA species contained 16 carbons in 3T3-L1 adipocytes and 18 carbons in L929 cells. These preliminary analyses suggested that

both cell lines could be useful to analyze metabolic changes during adipocyte differentiation. (**a**) L929 cells before and after differentiation in adipocytes. (**b**) 3T3-L1 cells before and after differentiation in adipocytes. (**c**) and (**d**) Comparison of the profiles in wild type and ECHDC1 KO L929 (**c**) and 3T3-L1 (**d**) cells. Arrowheads indicate the FA species that are increased in KO cells. Note that the tracing corresponding to the KO L929 cells is also shown in Fig. 3a. AU, arbitrary units, TIC, Total ion current.

Figure S5. ECHDC1 limits but does not completely prevent the formation of methyl-branched fatty acids in adipocyte models

Fatty acid methylesters derived from wild type or ECHDC1 knockout (KO) L929 and 3T3-L1 adipocytes were analyzed by GC-MS. (**a**)-(**d**) Representative EICs corresponding to FAs from L929 adipocytes with a total carbon number of 14, 15, 19 and 20 (m/z 242, 256, 312 and 326, respectively). (**e**)-(**k**) Representative EICs for FAs from 3T3-L1 cells with a total carbon number of 14 to 20 (m/z 242, 256, 270 ,284, 298, 312 and 326, respectively). Methyl-branched FA species are indicated in black. (**l**)-(**p**) Quantitative GC-MS analysis of monomethyl-branched FAs containing 17 carbons from wild type and ECHDC1 KO L929 adipocytes clones, as well as a KO clone transduced with a recombinant lentivirus driving the expression of ECHDC1 (“ECHDC1 cDNA”) or an empty vector (“Ctrl”). Non-separated species (represented in Fig. 3) were quantified together. Signals were normalized to the sum of all straight-chain FAs and are presented as means +/- SEM for at least 3 independent experiments. Asterisks indicate $p<0.05$ in post-hoc testing. rel.conc., relative concentration.

Figure S6. Identification by different GC-MS approaches of C17:0-carbon monomethyl-branched fatty acids derived from 3T3-L1 adipocytes.

(**a**) Representation of the 7 isomers of monomethyl-branched C16:0 expected to be formed by methylmalonyl-CoA incorporation. (**b**) Spike-in of C17:0 fatty acid methylesters (FAMEs) isomers as indicated (in bold) in extracts of ECHDC1 knockout (KO) adipocytes. The upper tracing is not spiked. SC Straight chain. (**c**)-(**i**) Assignment of the position of methyl-branches by spectra analysis according to Apon and Nicolaides (41). The vertical bars in the upper chromatogram (obtained from KO adipocytes) indicate the retention times at which the spectra shown in panels (d) to (i) were recorded. Circled values in the spectra are specific to the methyl-branched species indicated above each panel. (**j**) Molecular structure and expected fragmentation pattern of the 3-pyridylcarbinol (“picolinyl”) derivatives of C17:0 and 12-methyl-C16:0. The presence of a methyl-branch on C-12 of the latter makes that no m/z 290 ion is expected in the spectrum. (**k**) Representative EICs of “picolinyl”derivatives C17:0 FAs isomers in wild type and ECHDC1 KO adipocytes. The vertical bars indicate the retention times at which the spectra displayed in (**l**) were taken. Ions with decreased intensity (circled) indicate the position of the branch. SC, straight chain, AU, arbitrary unit.

Figure S7. Methyl-branched fatty acids are incorporated in phospholipids and sphingomyelin species

(**a**)-(**b**) LC-MS lipidomic analysis (negative electrospray ionization) of several phosphatidylethanolamine (PE) species containing methyl-branched FAs in wild type

(in white) and knockout (KO, in black) L929 adipocytes. PE(O-xx) refers to plasmanyl species and PE(P-xx) refers to plasmenyl species (plasmalogens). ‘xx’ indicates the total number of carbons in the fatty acyl or fatty alkyl moieties. (**b**) Representative EICs for PE(35:1) in wild type (WT) or ECHDC1 KO L929 adipocytes incubated without (upper tracings) or with (lower tracings) 2 mM d3-propionate. (**c**) LC-MS lipidomic analysis (positive electrospray ionization) of several sphingomyelin (SM) species containing methyl-branched FAs in wild type (white) and KO (black) L929 adipocytes. The “t” in SM(txx) refers to phytosphingosine (4-hydroxysphinganine). rel. conc. = concentration relative to total ion current; AU, arbitrary units.

Figure S1 (a to d)

a

Total carbons	Malonyl-CoA only	One methyl-malonyl-CoA	Two methyl-malonyl-CoA	Three methyl-malonyl-CoA	Four methyl-malonyl-CoA	Five methyl-malonyl-CoA	Six Methyl-malonyl-CoA
8	C8:0	absent	Di-C6:0	absent	absent	absent	absent
9	absent	Mono-C8:0	absent	absent	absent	absent	absent
10	C10:0	absent	Di-C8:0	absent	absent	absent	absent
11	absent	Mono-C10:0	Absent	Tri-C8:0	absent	absent	absent
12	C12:0	absent	Di-C10:0	absent	absent	absent	absent
13	absent	Mono-C12:0	absent	Tri-C10:0	absent	absent	absent
14	C14:0	absent	Di-C12:0	absent	Tetra-C10:0	absent	absent
15	absent	Mono-C14:0	absent	Tri-C12:0	absent	absent	absent
16	C16:0	absent	Di-C14:0	absent	Tetra-C12:0	absent	absent
17	absent	Mono-C16:0	absent	Tri-C14:0	absent	Penta-C12:0	absent
18	C18:0	absent	Di-C16:0	absent	Tetra-C14:0	absent	absent
19	absent	Mono-C18:0	absent	Tri-C16:0	absent	Penta-C14:0	absent
20	C20:0	Absent	Di-C18:0	absent	Tetra-C16:0	absent	Hexa-C14:0
21	absent	Mono-C20:0	absent	Tri-C18:0	absent	Penta-C16:0	absent

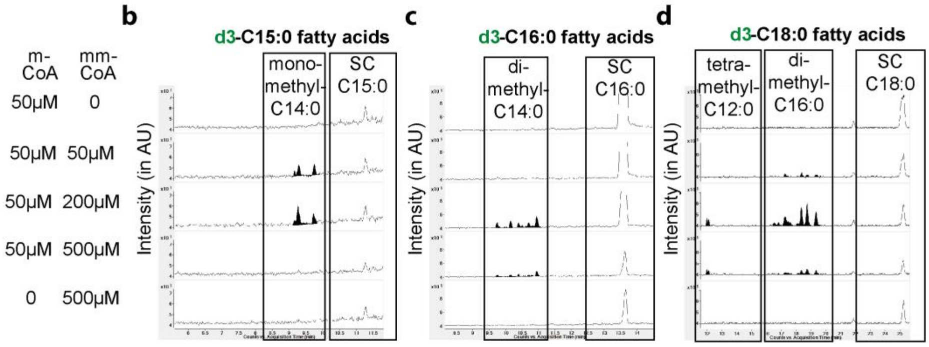

Figure S1 (e to k)

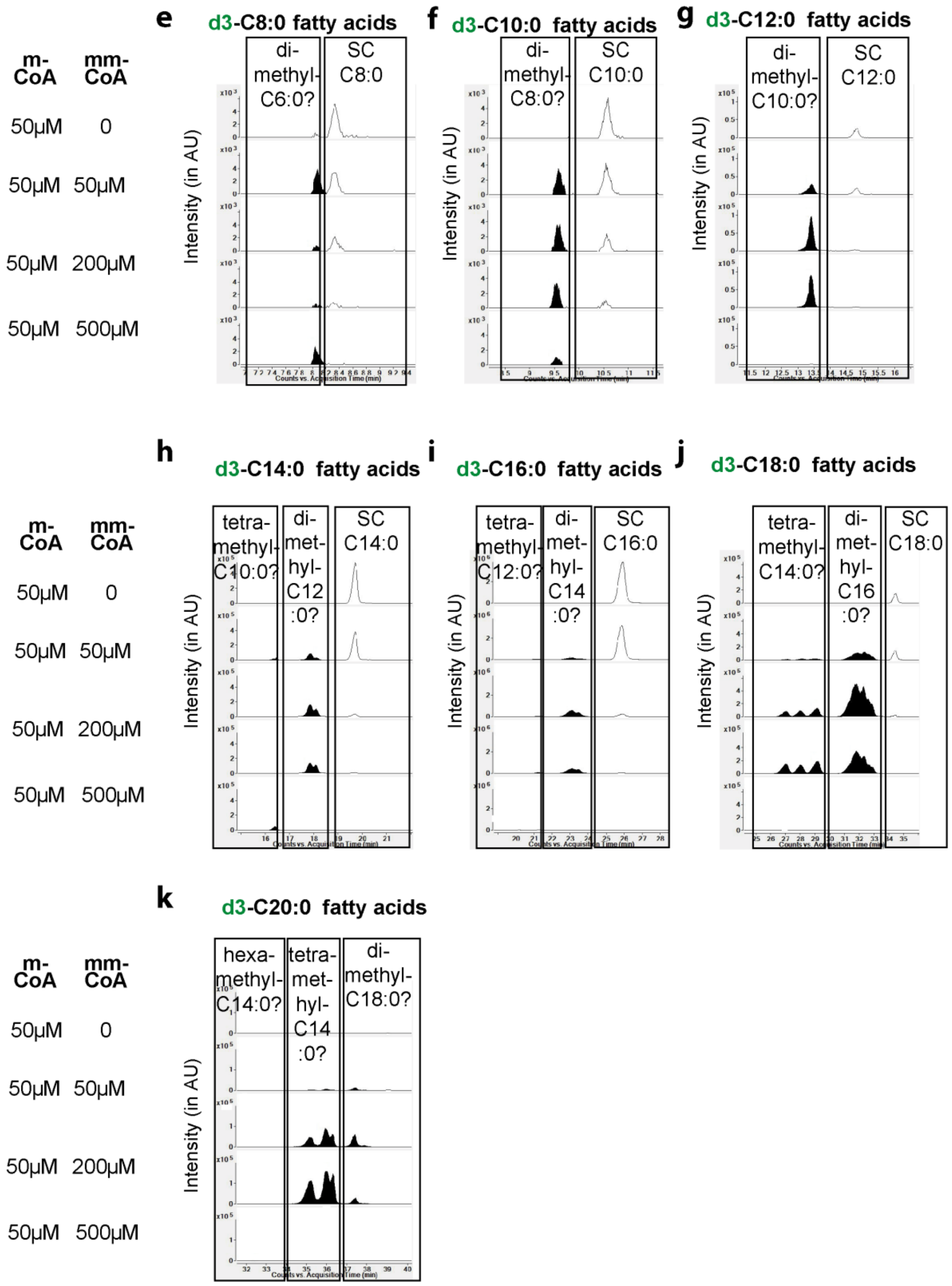

Figure S1 (l to r)

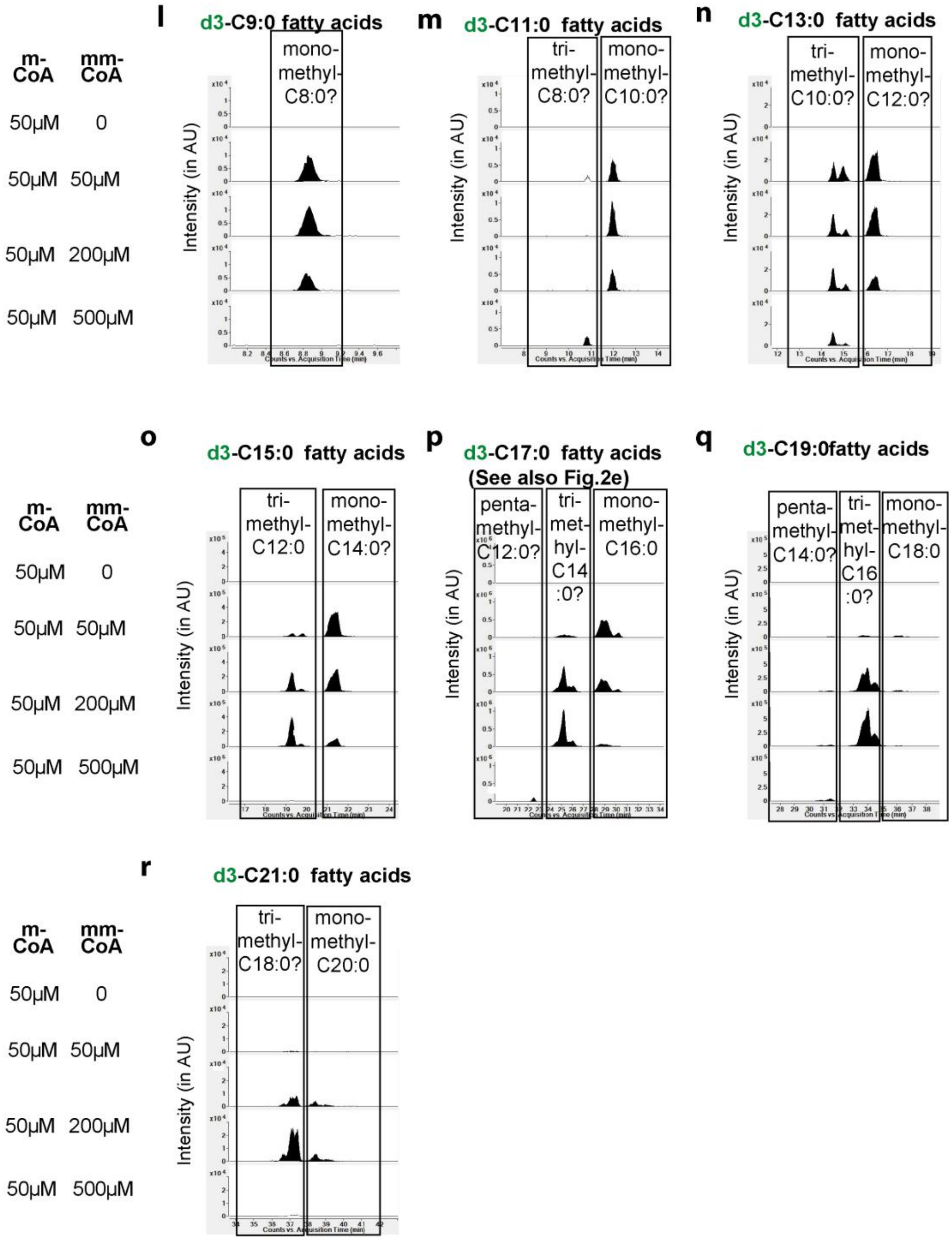

Figure S2

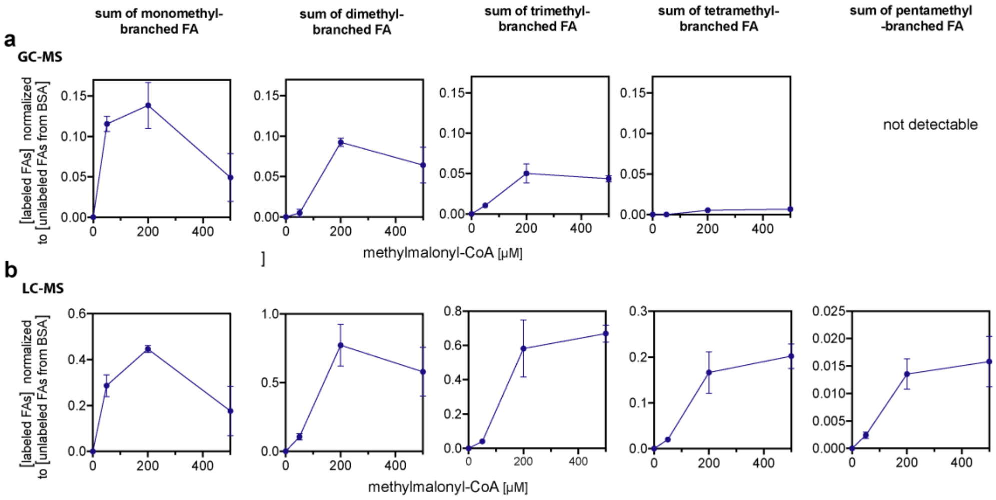
a
GC-MS
b
LC-MS
sum of monomethyl-branched FA
sum of dimethyl-branched FA
sum of trimethyl-branched FA
sum of tetramethyl-branched FA
sum of pentamethyl -branched FA
not detectable
[labeled FAs] normalized to [unlabeled FAs from BSA]
methylmalonyl-CoA [µM]

Figure S3

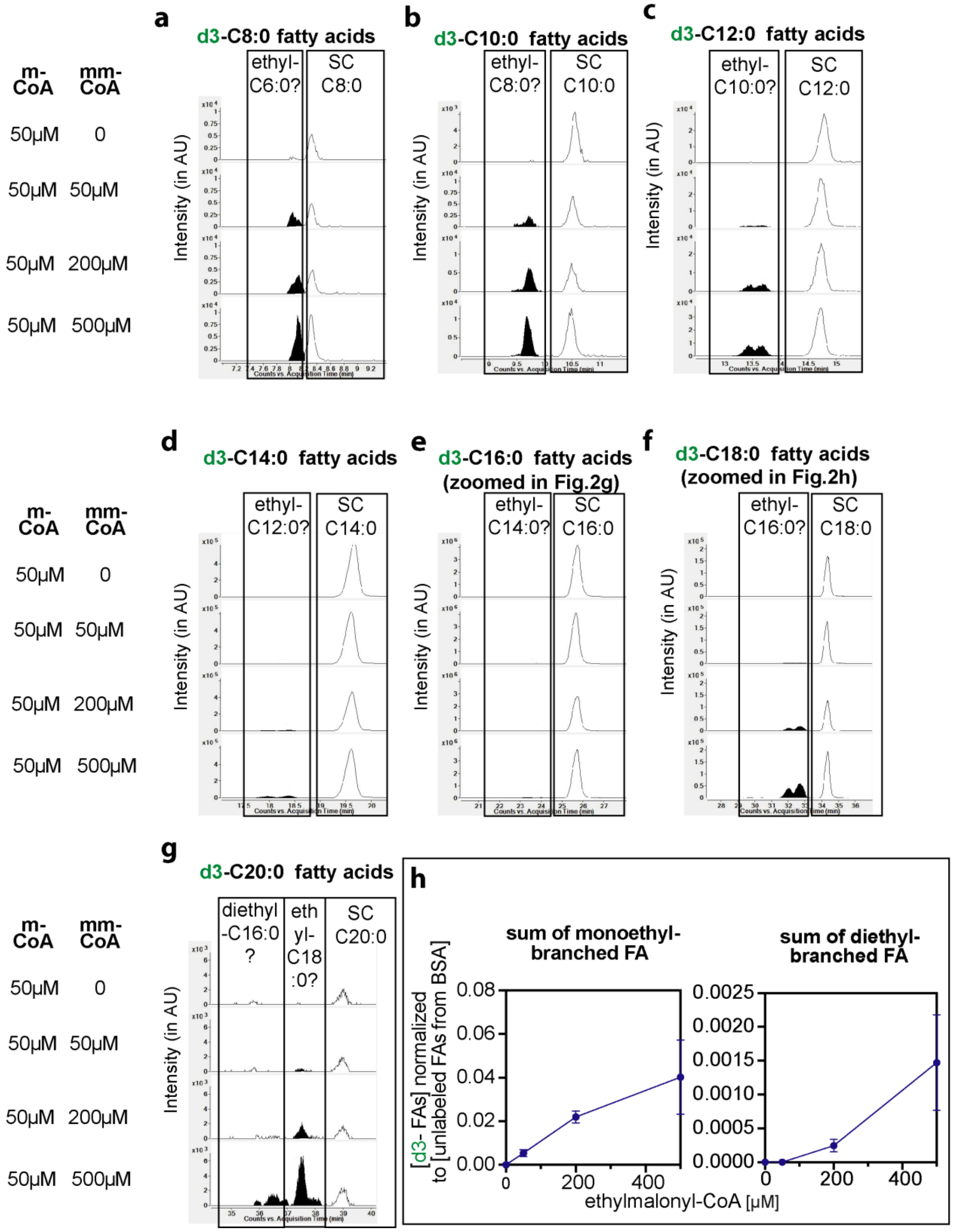

a
d3-C8:0 fatty acids
ethyl-C6:0?
SC C8:0
b
d3-C10:0 fatty acids
ethyl-C8:0?
SC C10:0
c
d3-C12:0 fatty acids
ethyl-C10:0?
SC C12:0
m-CoA
mm-CoA
50µM 0
50µM 50µM
50µM 200µM
50µM 500µM
Intensity (in AU)
d
d3-C14:0 fatty acids
ethyl-C12:0?
SC C14:0
e
d3-C16:0 fatty acids (zoomed in Fig.2g)
ethyl-C14:0?
SC C16:0
f
d3-C18:0 fatty acids (zoomed in Fig.2h)
ethyl-C16:0?
SC C18:0
g
d3-C20:0 fatty acids
diethyl-C16:0?
ethyl-C18:0?
SC C20:0
Counts vs. Acquisition Time (min)
h
sum of monoethyl-branched FA
sum of diethyl-branched FA
[d3- FAs] normalized to [unlabeled FAs from BSA]
ethylmalonyl-CoA [µM]
0.08
0.06
0.04
0.02
0.00
0.0025
0.0020
0.0015
0.0010
0.0005
0.0000
0
200
400

Figure S4

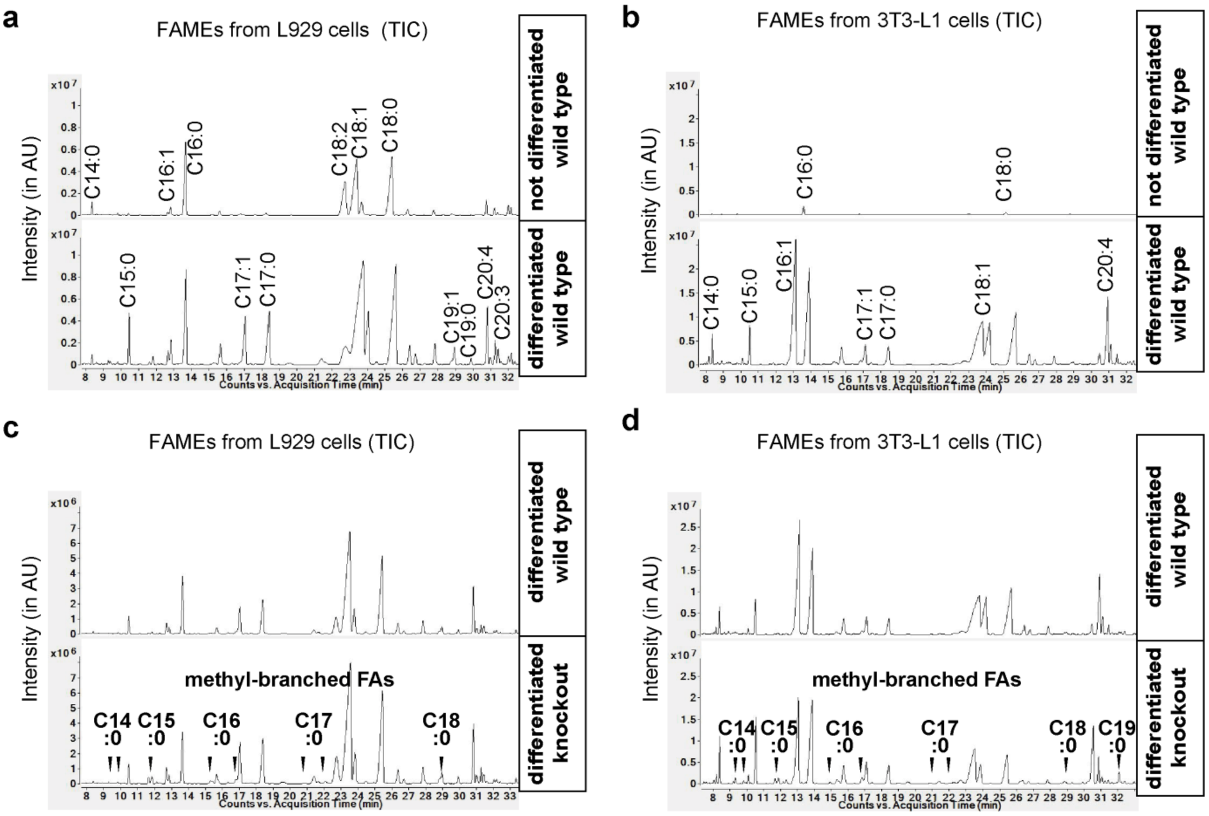

a
FAMEs from L929 cells (TIC)
not differentiated wild type
differentiated wild type
Intensity (in AU)
x10⁷
C14:0
C16:1
C16:0
C18:2
C18:1
C18:0
C15:0
C17:1
C17:0
C19:1
C19:0
C20:4
C20:3
Counts vs. Acquisition Time (min)
b
FAMEs from 3T3-L1 cells (TIC)
not differentiated wild type
differentiated wild type
Intensity (in AU)
C16:0
C18:0
C14:0
C15:0
C16:1
C17:1
C17:0
C18:1
C20:4
Counts vs. Acquisition Time (min)
c
FAMEs from L929 cells (TIC)
differentiated wild type
differentiated knockout
Intensity (in AU)
x10⁶
methyl-branched FAs
C14 :0
C15 :0
C16 :0
C17 :0
C18 :0
Counts vs. Acquisition Time (min)
d
FAMEs from 3T3-L1 cells (TIC)
differentiated wild type
differentiated knockout
Intensity (in AU)
x10⁷
methyl-branched FAs
C14 :0
C15 :0
C16 :0
C17 :0
C18 :0
C19 :0
Counts vs. Acquisition Time (min)

Figure S5 (a to d)

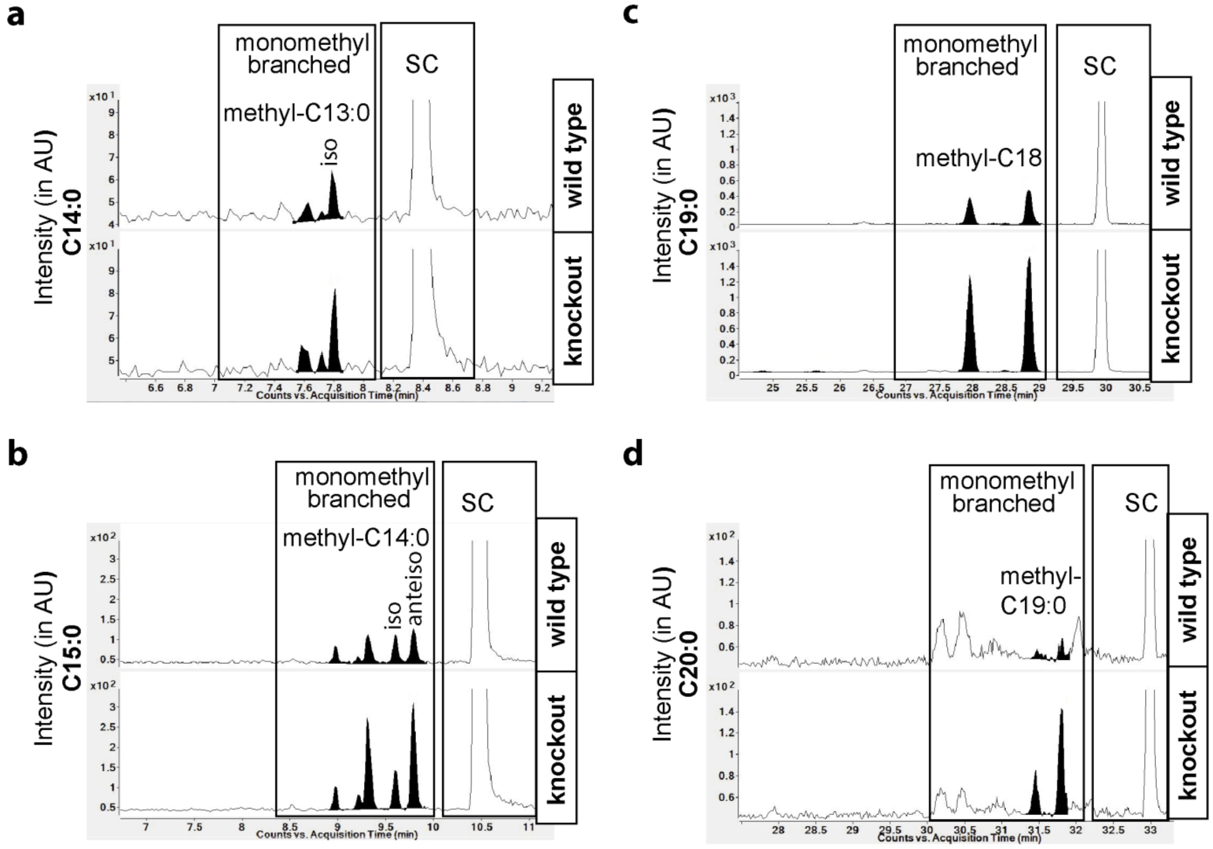

Figure S5 (e to k)

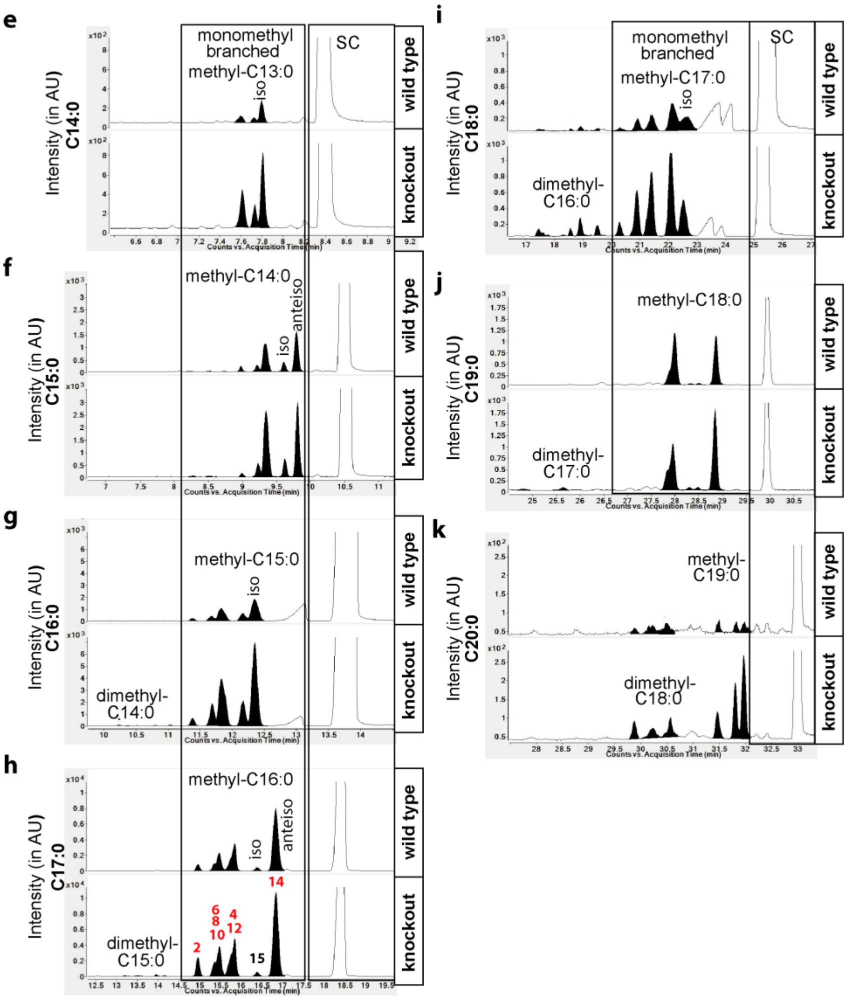

Figure S5 (l to p)

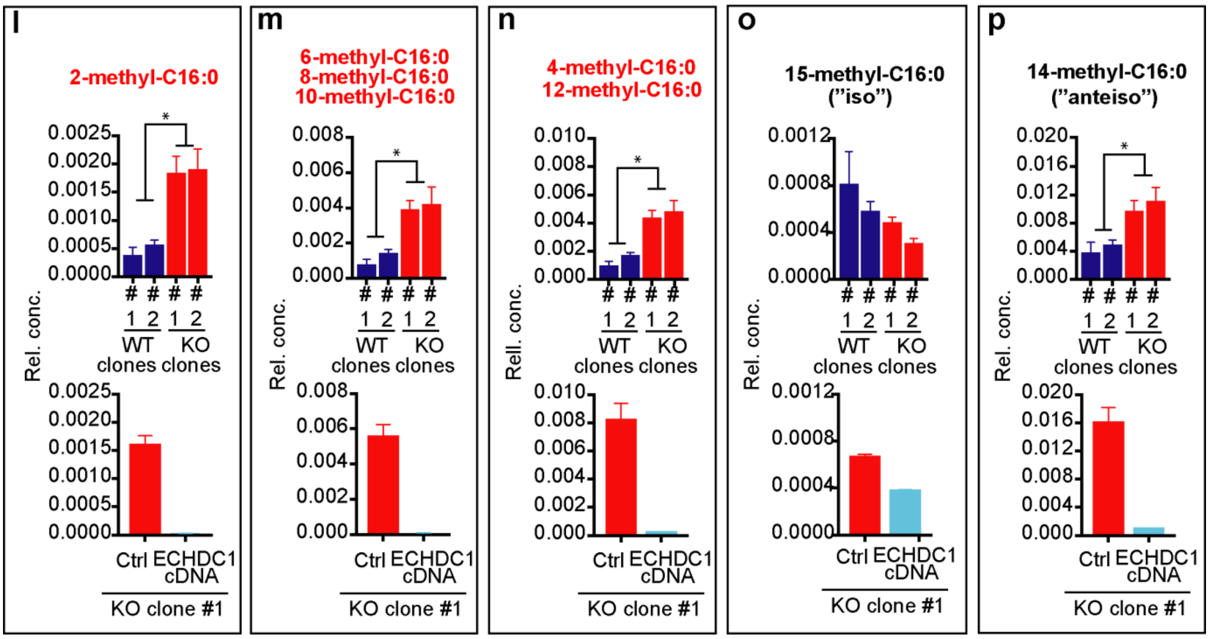

Figure S6 (a to b)

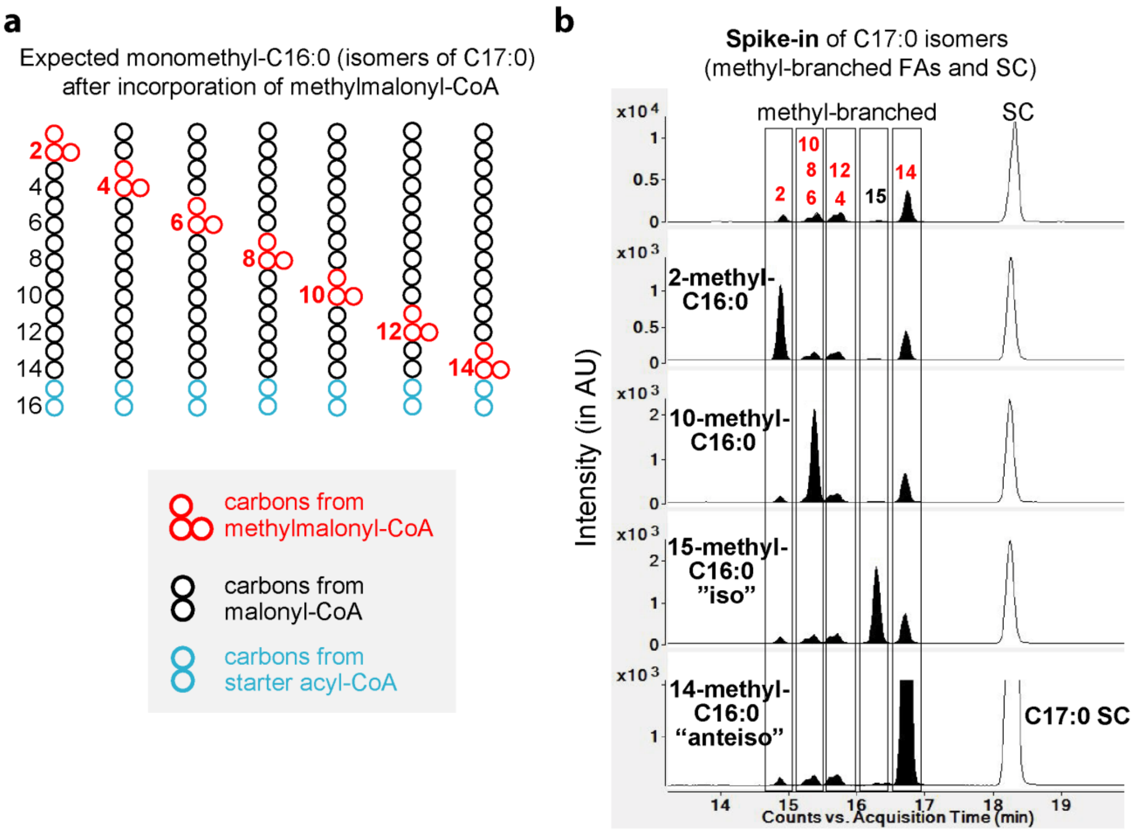

Figure S6 (c to i)

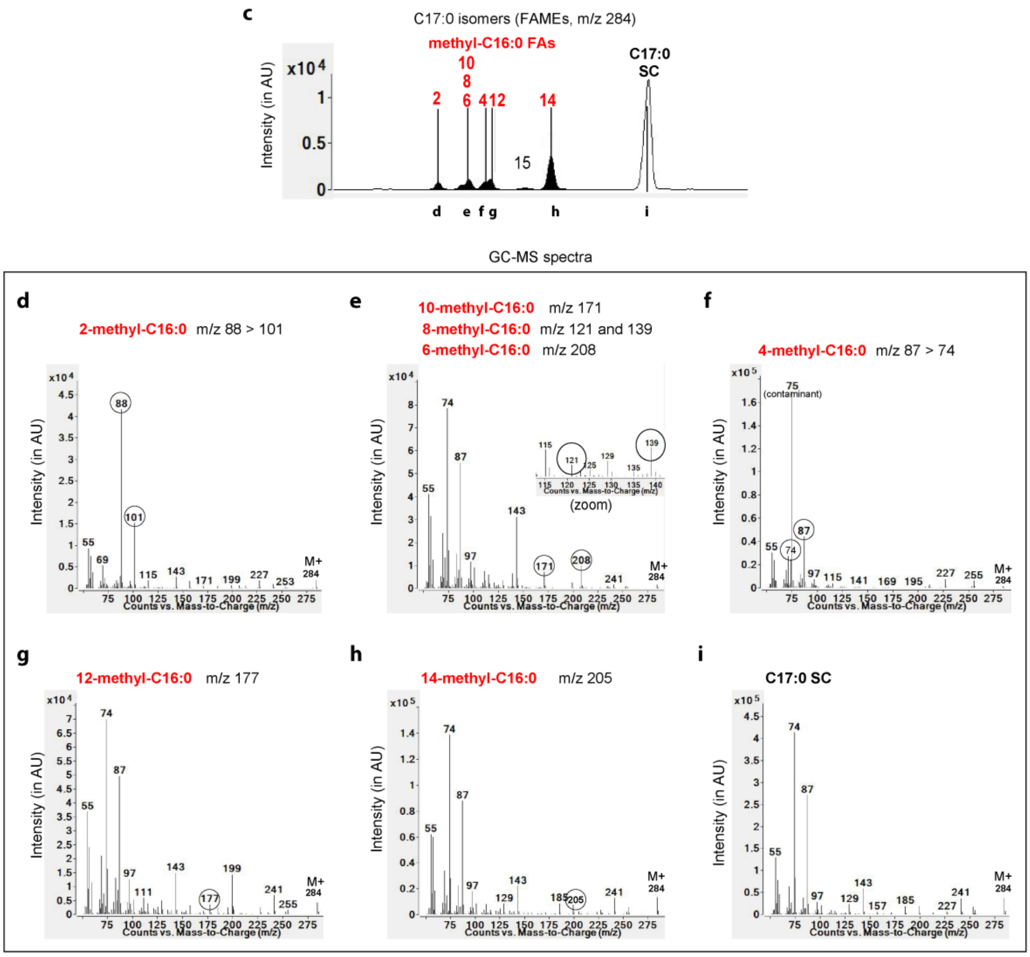

Figure S6 (j to l)

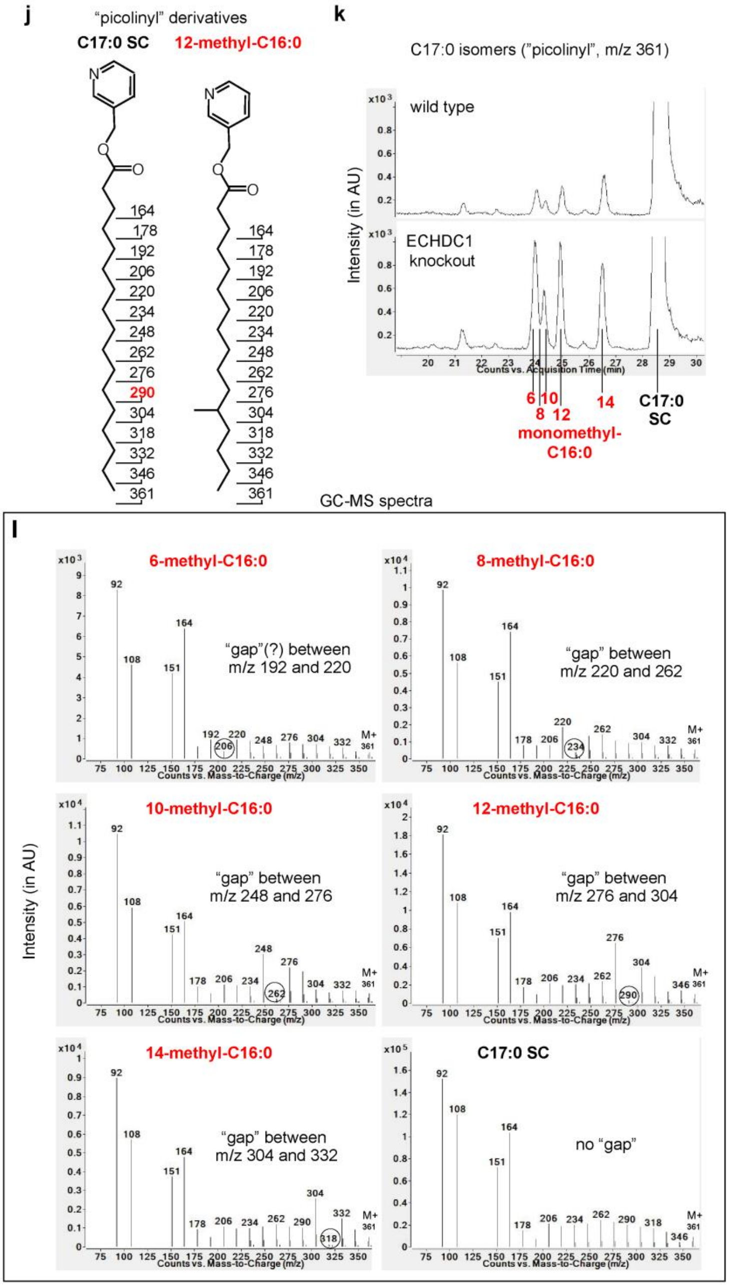

Figure S7

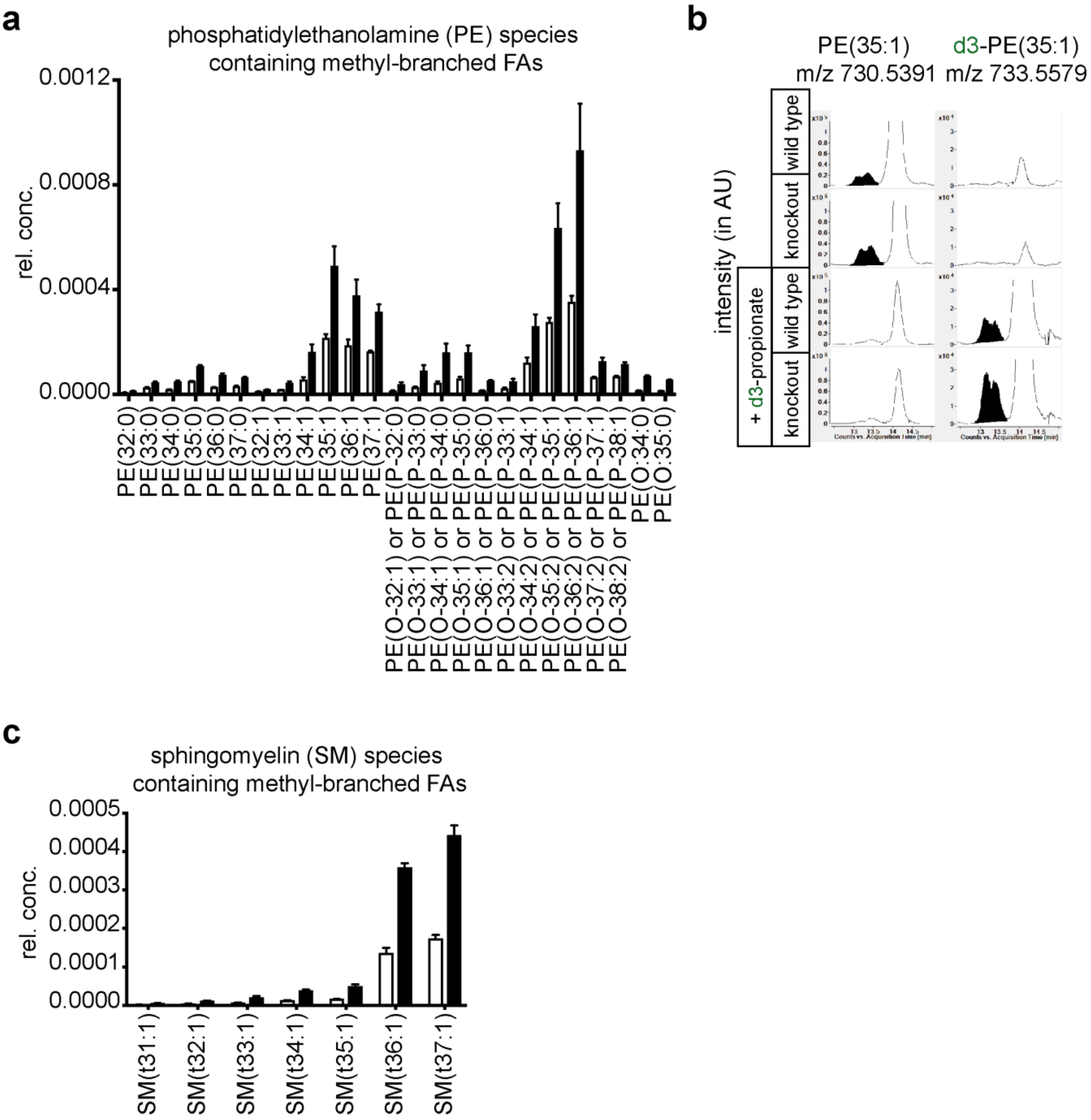
a
phosphatidylethanolamine (PE) species containing methyl-branched FAs
rel. conc.
0.0012
0.0008
0.0004
0.0000
PE(32:0)
PE(33:0)
PE(34:0)
PE(35:0)
PE(36:0)
PE(37:0)
PE(32:1)
PE(33:1)
PE(34:1)
PE(35:1)
PE(36:1)
PE(37:1)
PE(O-32:1) or PE(P-32:0)
PE(O-33:1) or PE(P-33:0)
PE(O-34:1) or PE(P-34:0)
PE(O-35:1) or PE(P-35:0)
PE(O-36:1) or PE(P-36:0)
PE(O-33:2) or PE(P-33:1)
PE(O-34:2) or PE(P-34:1)
PE(O-35:2) or PE(P-35:1)
PE(O-36:2) or PE(P-36:1)
PE(O-37:2) or PE(P-37:1)
PE(O-38:2) or PE(P-38:1)
PE(O:34:0)
PE(O:35:0)
b
PE(35:1)
m/z 730.5391
d3-PE(35:1)
m/z 733.5579
intensity (in AU)
wild type
knockout
+ d3-propionate
wild type
knockout
Counts vs. Acquisition Time (min)
c
sphingomyelin (SM) species containing methyl-branched FAs
rel. conc.
0.0005
0.0004
0.0003
0.0002
0.0001
0.0000
SM(t31:1)
SM(t32:1)
SM(t33:1)
SM(t34:1)
SM(t35:1)
SM(t36:1)
SM(t37:1)
